# Theoretical Screening of the Water Oxidation Electrocatalytic Cycle Promoted by Single-Site Macrocyclic Copper(II) Complexes: Unraveling the Role of the HPO_4_^2–^ Anion under Neutral Conditions

**DOI:** 10.1021/acsorginorgau.5c00047

**Published:** 2025-07-17

**Authors:** João Pedro C. S. Neves, Joel Leitão Nascimento, Bruno S. Sampaio, Roberto Rivelino, Tiago Vinicius Alves, Vitor H. Menezes da Silva

**Affiliations:** † Departamento de Físico-Química, Instituto de Química, 28111 Universidade Federal da Bahia, Rua Baraõ de Jeremoabo, Salvador, Bahia 40170-115, Brazil; ‡ Instituto de Física, 28111 Universidade Federal da Bahia, Salvador, Bahia 40210-340, Brazil

**Keywords:** water oxidation, 14-TMC ligand, electrocatalytic cycle, phosphate anion, O2 evolution, and DFT

## Abstract

Herein, a comprehensive theoretical investigation of the electrocatalytic cycle related to water oxidation (WO) (i.e., the bottleneck of H_2_ electrochemical production) catalyzed by a single-site copper­(II) complex bearing the 14-TMC ligand (1,4,8,11-tetramethyl-1,4,8,11-tetraazacyclotetradecane) is presented. Density Functional Theory (DFT) calculations were carried out to characterize two distinct single–electron transfer–water nucleophilic attack (SET-WNA) mechanisms that operate in the O–O bond formation in the electrocatalytic cycle. The electrochemical reaction is initiated with the formation of a [Cu­(14-TMC)­(H_2_O)]^+2^ intermediate, followed by a proton-coupled electron transfer (PCET) redox transformation. The first PCET is succeeded by a SET or a second PCET, leading to the formation of [Cu­(14-TMC)­(OH)]^+3^ (^4^ **2**) or [Cu­(14-TMC)­(O)]^+2^ (^4^ **3**), the starting reaction intermediates for the two SET-WNA mechanisms proposed. Notably, the presence of HPO_**4**_^**2–**^ plays a critical role in promoting the formation of the O–O bond formation. A proton transfer from water to HPO_4_^2–^ facilitates the O–O bond formation involving [Cu­(14-TMC)­(OH)]^+3^ (^4^2) and [Cu­(14-TMC)­(O)]^+2^ (^4^ **3**) species. To reinforce the crucial role of HPO_4_^2–^ under neutral conditions, in the absence of this anion, the overall reaction barrier for O–O bond formation is ≈40 kcal/mol, which rules out this reaction pathway at room temperature. Finally, successive PCET or SET steps involving [Cu­(14-TMC)­(HOOH)]^+2^ or [Cu­(14-TMC)­(OOH)]^+^ intermediates result in the evolution of the O_2_ and regeneration of the catalyst. The thermodynamic and kinetic computational results show good agreement with previous experimental electrochemical data. Overall, based on these computational findings, we propose a new picture for the WO electrocatalytic cycle mediated by macrocyclic Cu complexes bearing the 14-TCM ligand.

## Graphical abstract

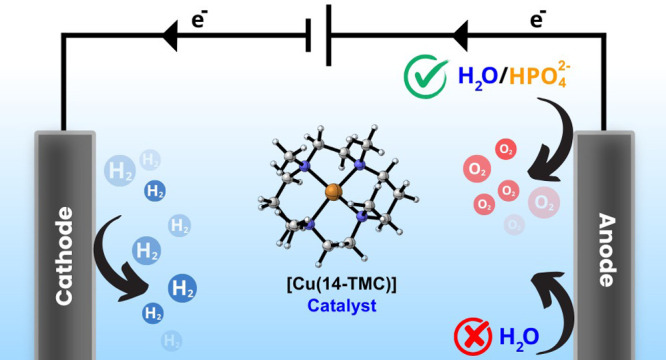

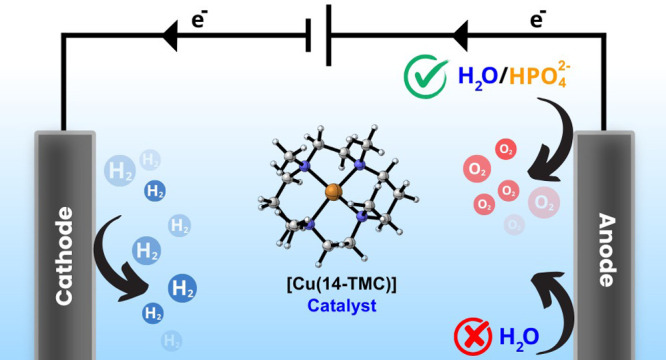

## 1 Introduction

Contemporary society faces challenges in achieving an economically efficient Energy Transition (ET). Today, the global economy remains dependent on nonrenewable fossil fuels, which are directly linked to increasing global temperatures and numerous catastrophic environmental consequences.^1,2^ Hydrogen gas (H_2_) has emerged as a promising green alternative to fossil fuels. Its exceptional energy density, due to the strong chemical bonds between hydrogen atoms, facilitates H_2_ for potential applications ranging from fuel cells to vehicles and industrial-scale processes.^3^ Most importantly, the use of H_2_ gas as fuel represents a clean and renewable alternative since it can be produced through sustainable processes such as solar-driven water electrolysis (eqs 1–3).^4,5^ Water electrolysis enables the conversion of an abundant natural resource into energetic (energy-dense) molecules, as occurs in photosynthesis.^5,6^

1 $$2H_{2}O\to O_{2}+2H_{2}$$

2 $$2H_{2}O\to O_{2}+4e^{‐}+4H^{+}\;E_{\text{SHE}}^{{}^{\circ}}=1.23\;V$$

3 $$4e^{‐}+4H^{+}\to 2H_{2}\;{\;E}_{\text{SHE}}^{{}^{\circ}}=0.00\;V$$

The major challenge of these electrochemical reactions lies not only in their thermodynamic demands but also in their kinetic limitations, necessitating the development of efficient catalytic alternatives.^7,8^ Specifically, the process bottleneck resides in O_2_ formation (eq 2), corresponding to the anodic half-reaction known as water oxidation (WO). During WO, chemical bonding between two oxygen atoms is achieved through the concerted release of four protons and four electrons from two water molecules, representing a mechanistically complex transformation. In recent decades, significant research efforts have focused on designing both homogeneous and heterogeneous catalysts to lower the activation energy barrier for WO, particularly for the O_2_ evolution step, to improve both the feasibility and scalability of this process.^9,10^

Inspired by biomimetic principles, metalloproteins are involved in natural redox processes^11–13^ that have inspired homogeneous transition metal complexes as potential catalysts for WO. Furthermore, valuable mechanistic insights have been derived from their well-explored catalytic cycles.^14,15^ The most promising catalytic performances have been achieved using noble metals (particularly Ir and Ru), despite their scarcity and high cost.^16,17^ In recent years, the balance between catalytic efficiency and economic viability has become crucial to achieve large-scale applications, placing first-row transition metals, such as Mn, Fe, Co, Ni, and Cu, in the research spotlight of WO catalysis.^18–20^ Notably, homogeneous Cu-based catalysts have recently emerged as particularly promising, primarily due to the natural abundance of copper and in specific its high lability,^21,22^ which allows facile ligand exchange and the dynamic formation and cleavage of bonds. This lability enables substitution by other ligands, as demonstrated in the work of Rudshteyn et al.,^23^ where the ligand dissociates to allow water coordination to the metal center for initiating the water oxidation process. A diverse range of coordination complexes were acting with various ligand architectures: from simple pyridines to peptide-type frameworks.^22–31^

In particular, Yu et al.^32^ synthesized a novel copper complex [Cu­(14-TMC)­(H_2_O)]­(NO_3_)_2_], where TMC is a macrocyclic ligand (14-TMC = 1,4,8,11-tetramethyl-1,4,8,11-tetraazacyclotetradecane) that demonstrated remarkable catalytic activity for WO catalysis. Under electrochemical conditions (0.1 M phosphate buffer) (applied potential = 1.40 V vs NHE, pH 7), this complex achieved WO activity. Kinetic studies based on electrochemical measurements indicate a turnover frequency (TOF) from a single-site WO with a value of 30 s^–1^, representing a significant WO rate for homogeneous copper catalysts under neutral conditions. Furthermore, cyclic voltammetry (CV) revealed two distinct redox waves at 1.64 and 1.77 V (versus NHE), which the authors attributed to consecutive proton-coupled electron transfer (PCET) and single-electron transfer (SET) processes, respectively (1.77 V peak is pH-independent over the range of measurements). Although there was no direct experimental evidence for the generation of high-valent Cu-based intermediates, these signals were tentatively assigned to Cu­(II)/Cu­(III) and Cu­(III)/Cu­(IV) processes, even though the oxidation of copper complexes to form high-valent Cu­(IV) should not occur easily. Despite the thorough experimental investigations, there remains a significant gap in understanding these electrochemical processes, depending on the nature of the ligand or the metal center.

It is well established in the literature that the formation of the O–O bond mediated by transition metal catalysts typically proceeds via two predominant mechanisms: (1) intermolecular coupling between two metal-oxo/oxyl species (I2M) or (2) water nucleophilic attack (WNA) on a high-valent metal-oxo intermediate. Under neutral solutions, the WNA mechanism initiates with a proton loss by water in order to drive the nucleophilic attack, while in alkaline conditions, the hydroxide ion (OH^–^) acts directly as the nucleophile, as illustrated in Scheme 1. In this context, the proposed WNA mechanism of Yu et al.^32^ has the O–O bond formation as the rate-determining step. Specifically, the [Cu­(14-TMC)­(HOOH)]^+2^ intermediate is formed from WNA followed by subsequent two-electron oxidation coupled with proton dissociation and O_2_ evolution.

**Scheme 1:**
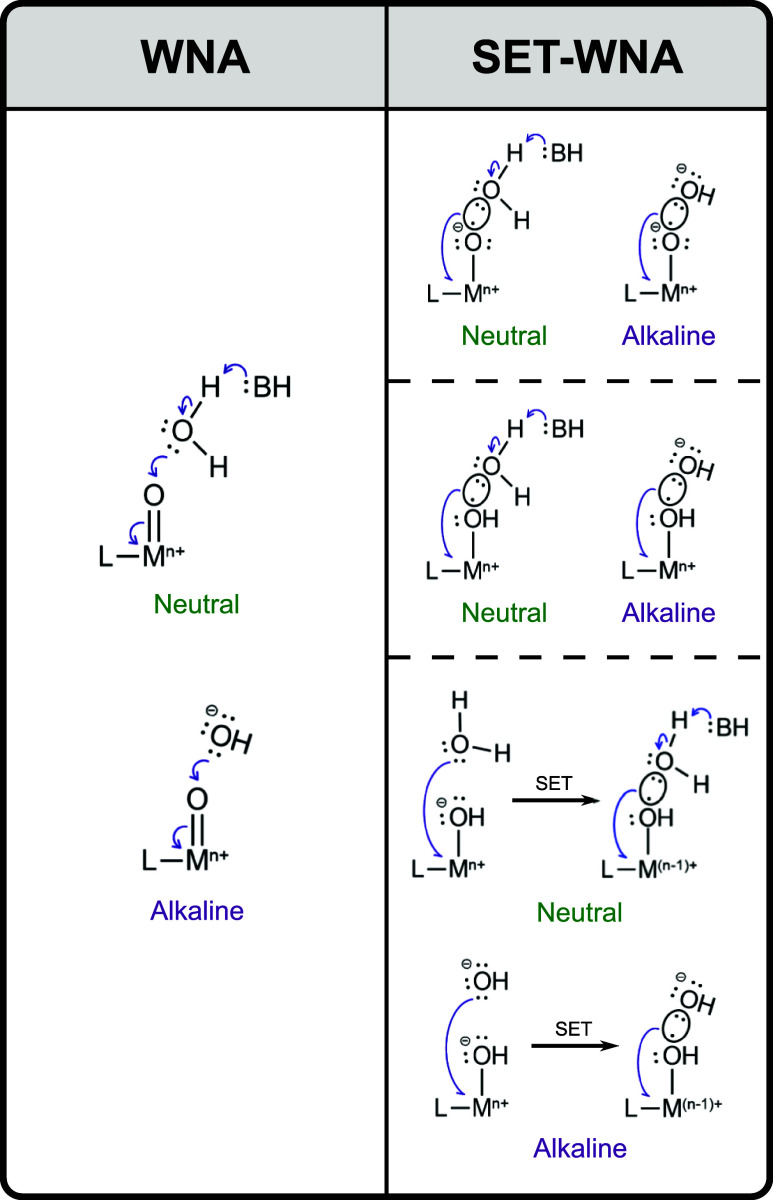
Differences between Water Nucleophilic Attack Mechanisms in Water Oxidation Catalysis with Transition Metal Complexes^a^

a Here, for a neutral solution, the proton abstraction and the WNA process may or may not occur in a concerted manner.

Recent computational studies of copper-based WO catalysts have proposed a new mechanistic scenario involving metal-mediated SET processes coupled to the WNA, in a process known SET-WNA.^33,34^ The two-step mechanism might be associated with the formation of an O–O bond through outer-sphere or inner-sphere SET processes from water or hydroxide. Furthermore, some computational studies have shown the crucial role of water-mediated interactions as a solvent in the O–O bond formation (use of the continuum solvation model with the inclusion of a limited number of water molecules to capture these interactions).^35,36^ More recently, anion–water interactions have also shown potential to facilitate the O–O bond formation involving Ni^37,38^ and Cu^36,39^ complexes. The interaction with a base such as HPO_4_^2–^ can facilitate the nucleophilic attack by water, especially under neutral pH, therefore serving as a key proton-accepting species.^40,41^ As outlined, as an alternative computational model, a cluster of water molecules may play perform the same role as a proton-accepting species.^35,36^

In this context, this computational study aims to enhance the understanding of the Cu-electrocatalytic WO reaction experimentally promoted by the single-site copper­(II) complex bearing the 14-TMC ligand,^32^ providing a comprehensive mechanistic analysis based on new theoretical findings. To achieve this goal, we systematically employed DFT calculations to explore the electrochemical transformations involving Cu complexes in different electronic states. Furthermore, this computational study was also performed to elucidate the reaction pathways associated with the WO catalytic cycle mediated by this Cu catalyst. Specifically, we propose a new mechanism for the formation of the O–O bond, the rate-determining step of the catalytic cycle. Indeed, the calculated free energy barrier agreed well with the experimental TOF, corroborating our new proposal for the WO electrocatalytic cycle involving Cu­(II) complexes bearing a macrocyclic 14-TCM ligand.

## 2 Methods

All calculations were based on the Density Functional Theory (DFT) as implemented in the Gaussian 09 program.^42^ Geometry optimization and frequency calculations of all stationary points (minima and transition states) were carried out using the density functional B3LYP-D3.^43,44^ The basis set def2-SVP^45^ was used for the lighter atoms, C, N, H, O, and P atoms, and in specific for Cu, the effective core potential basis set LANL2DZ­(f) was carried out.^46,47^ This electronic structure approach has been successfully employed for transition-metal-based WO electrocatalytic reactions.^33,36,39^ Furthermore, a good agreement was obtained using this computational approach to replicate selected experimental bond lengths (X-ray crystallography) of [Cu­(14-TMC)­(H_2_O)]­(NO_3_)_2_] obtained by Yu et al.^32^ (see Figure S1 and Table S1 in the Supporting Information). The electronic energies were refined through single-point corrections with M06L-D3 ^48^ using a larger basis set (def2-TZVP). This choice of M06L-D3 was supported by a benchmarking process (see Table S1), in which M06L-D3 accurately reproduces the experimental value of the first PCET (1.64 V).^32^ Additionally, previous studies have reported that M06L has good performance in describing organometallic/coordination complexes reactions^49,50^ including 3d metal transition-catalyzed water oxidation processes.^35,51,52^ Free energies in solution (water) were obtained using the SMD continuum-dielectric model^53^ in both geometry optimizations and single-point energy calculations. Open- and closed-shell calculations were performed for different electronic state structures by varying the total spin, resulting in multiplicity (1 for singlet, 2 for doublet, 3 for triplet, and 4 for quartet).

Intrinsic reaction coordinate (IRC)^54^ calculations were performed to confirm that each transition state was properly connected to the respective two minima. In the WO mechanism, an intersystem crossing of potential energy surfaces (PES) of different spin multiplicities is potentially expected. To address these critical points, relaxed scans were performed to identify these PES regions near the minimum energy crossing point (MECP). We also alternatively addressed the exact MECP using the EasyMECP code.^55^ Spin atom densities were evaluated for selected Cu complex structures using the Multiwfn program.^56,57^

The thermodynamics of the electrochemical processes involved in the WO catalytic process were evaluated by the redox potential (*E*°), estimated under standard conditions (pH = 0). The *E*° of PCET process is given by

4 $$E_{O|R}^{{}^{\circ}}=\frac{G^{{}^{\circ}}\left(O,\text{aq}\right)+n^{H+}G^{{}^{\circ}}\left(H^{+},\text{aq}\right)‐G^{{}^{\circ}}\left(R,\text{aq}\right)}{n_{e}F}‐E_{\text{SHE}}^{{}^{\circ}}$$

where *G*°(O, aq) and *G*°(R, aq) represent the Gibbs free energies of the oxidizing and reducing agents, respectively, and *G*°(H^+^, aq) is the free energy of the proton in solution, taken here as −270.3 kcal/mol (corresponding to the sum of its free energy solvation value in the gas phase (1 atm to 1 M) plus its experimental translational entropy in the gas phase).^58^ *E*_SHE_° stands for the potential of the standard hydrogen electrode (SHE), set at 4.281 V.^59^ The Faraday constant (*F*) was used as 23.08 kcal mol^–1^ V^–1^. The final Gibbs free energy corrections to 1 mol L^–1^ in water solution at room temperature were computed using the GoodVibes code,^60^ considering a frequency cutoff of 100 cm^–1^ and a correction for water concentration (55.56 mol L^–1^) of 4.27 kcal/mol.

To adjust the previously obtained redox potential reduction (*E*°) to the experimental conditions (pH = 7), we used the Nernst equations

5 $$E_{O|R}=E_{O|R}^{{}^{\circ}}‐\frac{n^{H+}}{n_{e}}\frac{RT}{F}\ln\left(10\right)\text{pH}$$

Since proton transfer processes occur in several steps of this WO catalytic cycle, the p*K*_a_ of Cu complexes acting as acids involved in these acid–base reactions was calculated by

6 $$pK_{a}=\frac{G^{{}^{\circ}}\left(A^{‐},\text{aq}\right)+n^{H^{+}}G^{{}^{\circ}}\left(H^{+},\text{aq}\right)‐G^{{}^{\circ}}\left(\text{AH},\text{aq}\right)+RT\ln\left(10\right)\text{pH}}{RT\ln\left(10\right)}$$

where A^–^ and AH are the base and acid species in solution, respectively.

Furthermore, for DFT calculations performed with iron-based complexes, counterions have been added to mitigate self-interaction errors,^61–63^ based on previous computational and combined computational/experimental studies on Cu-catalyzed water oxidation,^23,25,27,33–36,39^ where counterions were not included (i.e., the complexes were modeled as cationic or anionic species) and the experimental findings were still satisfactorily reproduced. Based on this evidence, we chose to neglect the counterions in our current study.

## 3 Results and Discussion

### 3.1 Electrochemical Activation of the Catalyst—PCET and SET Oxidation Process

It is established in the literature that before O–O bond formation, the crucial step in the WO catalytic cycle, oxidative activation reactions of the initial complex (catalyst) are necessary.^64^ Experimentally, Yu et al.^32^ observed two distinct electrochemical events indicated by the CV waves before the formation of the O–O bond. The first process is likely a PCET process since the respective wave showed a pH dependence during the experiment. DFT calculations were employed in order to estimate the redox potential of this PCET, which was computed to be 1.49 V, demonstrating good agreement with the experimental value (1.64 V). Regarding the second wave, it is probably associated with a SET process because of its lack of pH dependence. Despite that, we have also investigated the possibility of a second PCET, with a redox potential computed to be 1.72 V. Alternatively, as experimentally suggested, the SET redox potential was computed to be 1.66 V. Overall, both theoretical redox potential values are possible (experimental value of 1.77 V).

Furthermore, DFT calculations suggest the SET process as thermodynamically favorable in relation to a second PCET. These results are summarized in Figure 1. As evidenced, based on the reaction model under experimental conditions (a reference potential of 1.4 V vs NHE and a pH of 7.0), these oxidation processes are slightly endergonic. Initially, the catalyst [Cu­(14-TMC)­(H_2_O)]^2+^ (labeled **0**), which is a doublet in its electronic ground state, undergoes first a PCET that results in **1**. The resulting species from this oxidation is triplet (open-shell complex) ^3^ **1**, which is more stable than singlet ^1^ **1** by 3.52 kcal/mol. Figure 1 shows that the formation of ^3^ **1** is smooth^20^ in this applied potential. Spin density calculations (see Figure S2 in the Supporting Information) showed that electron density loss is spread over the molecular structure, with contributions of oxygen and copper, and most expressively on the ligand, showing the redox activity of 14-TCM.

**Figure 1:**
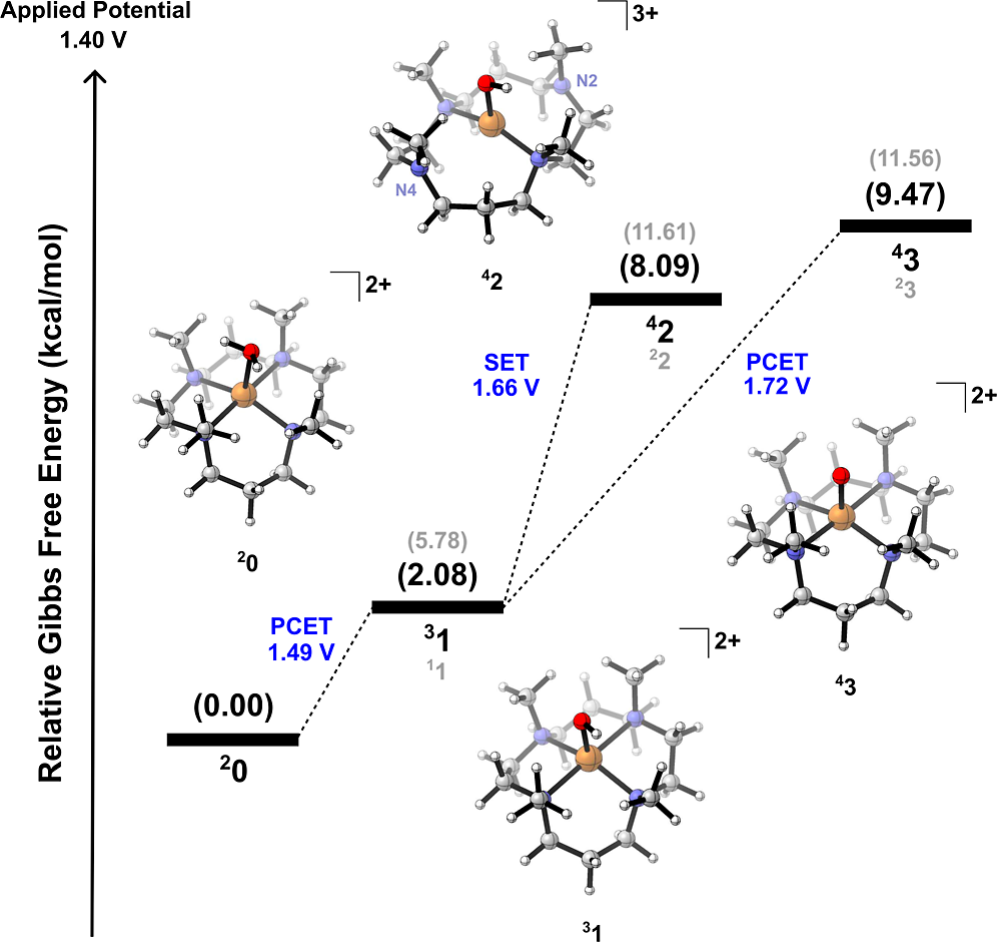
Gibbs Free energy (298.15 K) diagram derived from experimental conditions for the SET and PCET oxidation processes. Redox potential values (pH = 7) corresponding to these oxidation steps were shown in blue.

Following the second oxidation process on ^3^ **1**, the resulting complex of the SET is ^4^ **2** (the quartet ^**4**^ **2** is more stable than doublet ^2^ **2** by 3.52 kcal/mol). Taken together the optimized geometries and spin densities of ^4^ **2**, it is possible to perceive that both nitrogens (labeled N2 and N4) are uncoordinated and each started to hold one unpaired electron with a formal charge of +1 (see section S3 of Supporting Information for a detailed analysis of this complex). This is possible due to the conformation flexibility of 14-TCM and lability of the Cu­(II) center,^65^ which reinforces the redox activity of the ligand. Indeed, both copper and oxygen did not exhibit substantial shifts in the spin density.

On the other reaction path, the second PCET (**1** → **3**), DFT calculations suggest the quartet as the most stable state (by 2.09 kcal/mol) compared to the doublet. Spin density population analysis shows in this case that there is an electron loss on the oxygen atom, suggesting that the nature of the Cu–O bond is not a double bond as expected for a Cu=O oxo-complex. This can be rationalized because the Cu metal is situated far beyond the “oxo wall”, as it is well demonstrated in the literature.^34,66–68^ More specifically, this states that in transition metal complexes with square-pyramidal geometry and d^*n*^ orbitals with *n* ≥ 5, the occupation of π* orbitals decreases the double-bond character. As a result, it is more reasonable to describe **3** by its Cu-oxyl radical nature.

### 3.2 Mechanism of the O–O Bond Formation

According to Scheme 1, given the experimental conditions (pH = 7), after either a SET (**1** → **2**) or a second PCET (**1** → **3**), the O–O bond formation might be related to the nucleophilic attack of a water molecule on the oxygen coordinated to the Cu complex. As suggested in previous computational studies on the WO mechanism, a proton abstraction is expected to be assisted by other species in solution. Unfortunately, we were not able to find a transition state for the O–O bond formation using water clusters (mixed cluster-continuum approaches), even using three or four water molecules.^35,36^ Alternatively, our calculations show the role of the HPO_4_^2–^ anion from pH phosphate buffer acting on the O–O bond formation. Figure 2 reveals the free energy diagrams associated with the presence of HPO_4_^2–^ in the O–O bond formation through ^**4**^ **2** (a) and ^**4**^ **3** (b).

**Figure 2:**
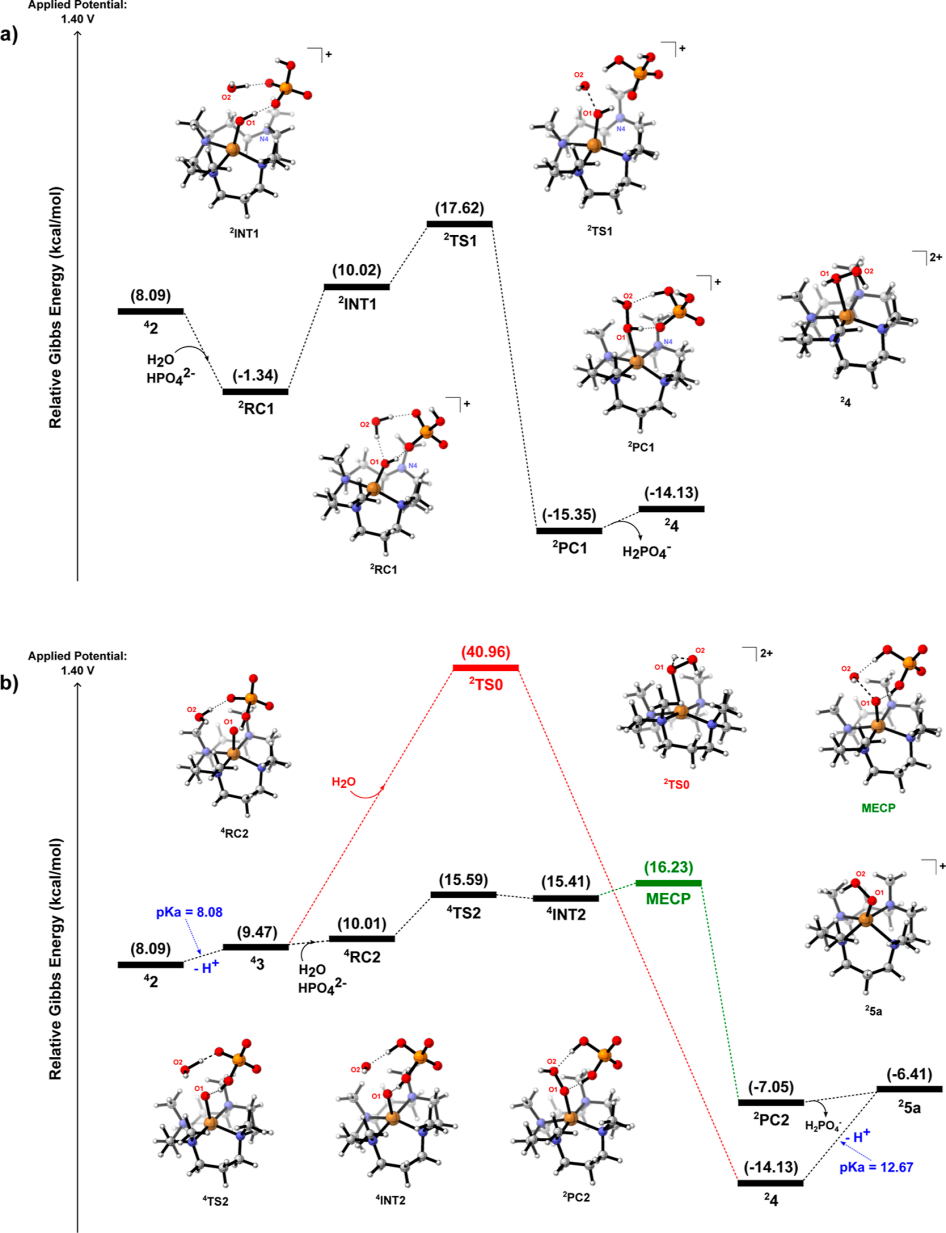
Gibbs free energy (298.15 K) diagram for the O–O bond formation mechanism takes into account the process from either (a) SET (**1** → **2**) or (b) second PCET (**1** → **3**).

#### 3.2.1 SET Mechanism from Intermediate **2**

Taking our attention first to the reaction pathway from intermediate ^**4**^ **2** (Figure 2a), it is possible to notice the water molecule approximating the HPO_4_^2–^ ion in the Cu vicinity. After that, two possible doublet prereactive complexes were identified by the calculations: the first one, ^**2**^ **RC1**, which is 9.43 kcal/mol more stable relative to ^**4**^ **2**, and the second one ^**2**^ **INT1**, 1.94 kcal/mol less stable. Both exhibit the copper center with a four-coordination number and a geometry resembling a distorted tetrahedron. Specifically, in their structure, one nitrogen is apart (uncoordinated) from the metallic ion. Subsequently, a transition state with doublet multiplicity was located (^**2**^ **TS1**). The IRC calculations of ^**2**^ **TS1** reveal ^**2**^ **RC1** as the backward intermediate in the reaction coordinate. However, the spin density population depicted in Figure 3 suggests that complex ^**2**^ **RC1** could not be directly connected to ^**2**^ **TS1** since it would be described as a WNA mechanism. However, this one may be discarded due to the absence of a typical oxo-complex wherein the Cu=O bond receives the nucleophilic attack of water. Besides, on ^**2**^ **RC1**, the oxygen labeled as O1 is not an appropriate electrophilic site as a consequence of its formal negative charge (see this in detail in Figure 3). Interestingly, in ^**2**^ **INT1** geometry, the electron pairs of the oxygen atom O2 are directed toward the other oxygen atom O1. This situation reinforces that a two-step SET-WNA mechanism seems to be operative to the O–O bond formation involving ^**2**^ **INT1** formation as the initial step.

**Figure 3:**
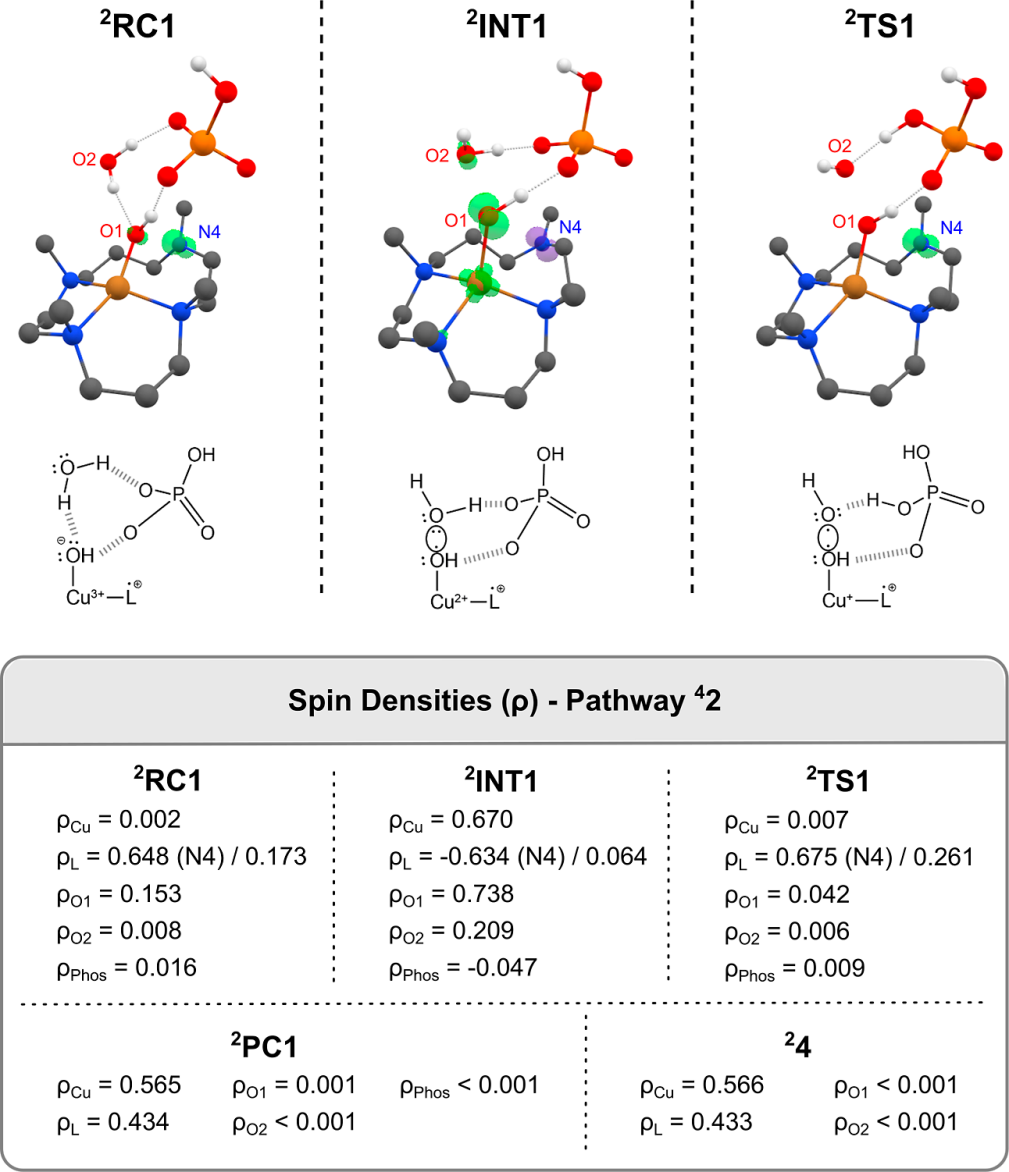
Spin densities of species involved in the ^**4**^ **2** Pathway; H atoms attached to carbon were omitted for clarity.

Analyzing more deeply the structure of ^**2**^ **RC1**, Figure 3 depicts the Cu with a spin density of 0.002 and, therefore, an oxidation state of +3. Based on that, the first step of the O–O bond formation is possibly a SET from the water molecule to the Cu center, leading to the intermediate ^**2**^ **INT1** with three unpaired electrons, as indicated in Figure 3 by the variations in spin density population from ^**2**^ **RC1** to ^**2**^ **INT1**. Indeed, in ^**2**^ **INT1**, the metallic ion manifests an oxidation state of +2 due to the gained electron. In addition, the remaining unpaired electron on the oxygen of water forms a weak covalent bond between the oxygen atoms (a three-electron O–O bond). This slightly repulsive type of bonding was predicted by Pauling;^69^ it can be seen when one unpaired electron occupies an antibonding orbital associated with these respective atoms,^70,71^ possibly explaining the higher energy of ^**2**^ **INT1** relative to ^**2**^ **RC1**. Unfortunately, we could not locate a transition state of this inner-sphere SET process,^72^ possibly due to geometric similarities between ^**2**^ **RC1** and ^**2**^ **INT1**, which suggests a not-so-appreciable reaction barrier for this step, thus supporting ^**2**^ **TS1** to be responsible for the TOF observed in this WO catalysis. Based on that, we believe that ^**2**^ **RC1** should overcome ^**2**^ **INT1** on the free energy profile as the thermodynamic driving force for ^**2**^ **PC1** formation.

The optimized structure of ^**2**^ **TS1** reveals the formation of the O–O bond with an imaginary frequency of 209.10*i* cm^–1^. As outlined, ^**2**^ **TS1** is characterized as the final step of the two-step inner-sphere SET mechanism. Indeed, the spin density analysis population indicates another electron gained by the copper atom (resulting in an oxidation state of +1) from the three-electron O–O bond of ^**2**^ **INT1** (Figure 3). It is interesting to notice that the O–O bond formation happens along with the proton abstraction by the HPO_4_^2–^ ion, which was confirmed by the IRC calculations. This is consistent with a possible atom-proton transfer process in the formation of the O–O bond, as experimentally evidenced by using kinetic isotope effect studies. This step is considered to be the rate-determining step with a free energy barrier of 17.62 kcal/mol relative to the catalyst. Based on the energetic span model and considering ^**2**^ **TS1** and ^**2**^ **RC1** as the TDTS and TDI energy states, respectively, the TDTS-TDI^73,74^ energy difference is 18.96 kcal/mol. This value is close to the corresponding experimental free energy barrier derived from the experimental TOF (30 s^–1^) using the Eyring equation (15.2 kcal/mol). Finally, the IRC forward scan led to the final complex of this pathway, labeled ^**2**^ **PC1**, wherein the five-coordinate environment is recovered. According to spin density insights, both ligand and copper hold the remaining unpaired electrons. Finally, the H_2_PO_4_^–^ ion departs from the system, resulting in ^**2**^ **4**.

#### 3.2.2 SET Mechanism from Intermediate **3**

DFT calculations support the role of the HPO_4_^2–^ ion into the O–O bond formation. In order to compare the absence of HPO_4_^2–^ (having only the water molecule acting in this process), we calculated an alternative reaction route (^**2**^ **TS0**). In this route, after the formation of the O–O bond, one of the hydrogens of oxygen, O2 goes to O1 (see details of this in the ^**2**^ **TS0**-optimized geometry depicted in Figure 2b). This atom transfer is well captured by the vibrational mode associated with the imaginary frequency of ^**2**^ **TS0**. Furthermore, IRC calculations on ^**2**^ **TS0** have shown that this process is concerted with respect to the connection between the oxygen atoms to form the new O–O bond. Unfortunately, we were not able to locate a specific transition state for this final step, although we attributed the completion of the O–O bond probably through an inner-sphere SET from the three-electron O–O bond between the oxygens (O1 and O2), likewise discussed in the HPO_4_^2–^-assisted mechanism from **2**. The existence of this SET can explain the shift in spin multiplicities from ^**4**^ **3** to ^**2**^ **TS0**. Based on that, the route under discussion takes place in a doublet mechanism. Indeed, spin density population analysis indicates one unpaired electron held by the complex in ^**2**^ **TS0** (and thus a metal center charge of +2), while in ^**4**^ **3**, there are two unpaired electrons on the Cu (+3 charge) and another unpaired electron localized on the oxygen. However, the resulting barrier is given by 40.96 kcal/mol, demonstrating the prohibitive nature of this mechanism under the current experimental conditions (room temperature). In that sense, these computational insights support the HPO_4_^2–^-assisted mechanism as much more favorable.

As depicted in Figure 2b, another possible pathway of assisted O–O bond formation arises from ^**4**^ **3**. We considered investigating the viability of this route to extend the mechanistic perspective of the electrocatalytic cycle, comparing it to other computed reaction paths shown here. This reaction path begins with the reactive complex ^**4**^ **RC2**. We were able to find a transition state (^**4**^ **TS2**) associated with the abstraction of a proton from water by the HPO_4_^2–^ ion. This was confirmed by the visualization of vibration mode related to the imaginary frequency of ^**4**^ **TS2** and further supported by IRC calculations of its structure. Based on that, it has confirmed ^**4**^ **RC2** as the initial reactive complex, also pointing out ^**4**^ **INT2** as the resulting product of this step. In addition, by analyzing the spin densities along this quartet pathway (Figure S2), no significant changes are evidenced from ^**4**^ **RC2** to ^**4**^ **INT2**. It is worth mentioning that, despite our efforts, we were unable to locate doublet analogues to the quartet species discussed in this particular case. As shown in Figure S3, these three quartet structures feature a copper center with a charge of +2 and a double-radical localized on oxygen O1 (with one of the unpaired electrons being shared with O2 in a three-electron bond, in a similar fashion observed in ^**2**^ **INT1**).

Despite our efforts to locate a quartet TS for the formation of an O–O bond, it was unsuccessful, as the system exhibited repulsive behavior along the O–O bond direction. On the other hand, similar attempts with doublet multiplicity promptly led to the connection of oxygen atoms, albeit without the localization of TS as a stationary point on the potential energy surface. We verify that ^**2**^ **PC2** is localized with the complete formation of the O–O bond. We wonder whether an intersystem crossing will emerge in this case. To investigate this subject, we performed a relaxed energy scan using ^**2**^ **PC2**, varying the O–O bond distance. We ranged the distances in the direction of the breaking of the O–O bond and the O–O bond distance of ^**4**^ **INT2** in the direction of the forming of the O–O bond; a step size of 0.03 Å was used. Figure 4 shows the graph related to this energy scan along the O–O reaction coordinate.

**Figure 4:**
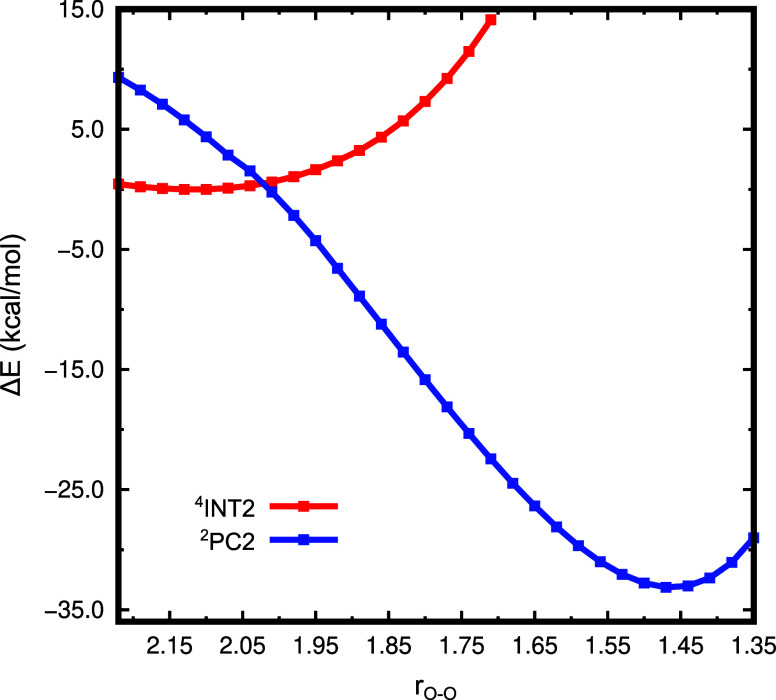
Potential energy relaxed scan along the O–O reaction coordinate for ^**2**^ **PC2** and ^**4**^ **INT2**.

Interestingly, it is possible to notice a potential crossing between the surface’s doublet and quartet around 1.95 Å–2.05 Å. Using Harvey’s algorithm^75^ implemented in the EasyMecp code,^55^ we located the minimum energy crossing point (referenced by MECP in Figure 2b) at 16.23 kcal/mol, with an O–O bond length of 1.998 Å.

When analyzing Voronoi and Hirshfeld atomic population charges^56^ on species ^**4**^ **INT2** and ^**2**^ **PC2** (Table S3), a reversal trend has been observed between oxygen O1 and O2. In addition, the spin density analysis based on the independent-particle picture (Figure S3) also suggests that this particular conversion from quartet to doublet state occurs through an electronic rearrangement. More specifically, this electronic spin-state conversion takes place via a migration of the unpaired electron from the σ_O–O_* orbital, resulting in pairing with the singly occupied orbital centered on the orbital of O1. Consequently, this shift of electronic configuration (^**4**^ **INT2** → ^**2**^ **PC2**) is favorable due to the existence of a lone-pair on O1, which thus eliminates the antibonding character of the O–O bond in ^**2**^ **PC2**.

Consequently, in the final step, the ^**4**^ **2** complex releases the H_2_PO_4_^–^ ion, resulting in the formation of ^**2**^ **5a**. Overall, these computational analyses led us to conclude that the potential intersection between the doublet and quarter energy surfaces is feasible and does not rule out this mechanism to be plausible. In addition, DFT calculations also revealed an alternative pathway for the formation of the ^**2**^ **PC2** species (see Figure S6 in Supporting Information). In this case, a new reaction pathway was identified on the doublet state, proceeding through a concerted transition state (^**2**^ **TS3**) located at 23.49 kcal/mol. Even though this transition state is energetically less favorable compared to both ^**2**^ **TS1** and MECP, this mechanism cannot be completely ruled out.

#### 3.2.3 O_2_ Departure and Catalyst Regeneration

The resulting O–O bond culminates in intermediates ^**2**^ **4** or ^**2**^ **5a**, which are connected by a proton loss with p*K*_a_ = 12.67. As depicted in Figure 5, both paths are going downhill on the free energy profile, i.e., strong exergonic electrochemical steps, both resulting in the departure of the O_2_ molecule together with the recovery of the catalyst. Starting from ^**2**^ **4**, two PCETs lead to ^**4**^ **8** as the resulting complex expected from this redox process. However, we expand the investigation on the departure of O_2_ by scrutinizing potential new reaction intermediates. That being so, both PCETs can be dismembered in a proton loss followed by a SET, where the first one (**4** → **6**) can have a proton loss on either O1 or O2. These possible chain steps are summarized in Figure 5. In the whole process, the spin densities show no significant change in the oxidation state of the copper center, which remains in +2. Instead, O1 and O2 are doubly oxidized (as well as deprotonated), first to the superoxide species ^3^7 and then to ^**4**^ **8**, where each oxygen has one unpaired electron. The ^**4**^ **8** structure displays an uncoordination between the copper and oxygen atoms (Cu–O distances of 3.77 and 3.64 Å). Lastly, to recover ^**2**^ **0**, triplet O_2_ is released from ^**4**^ **8** followed by the return of a water molecule to the complex to form the catalyst again.

**Figure 5:**
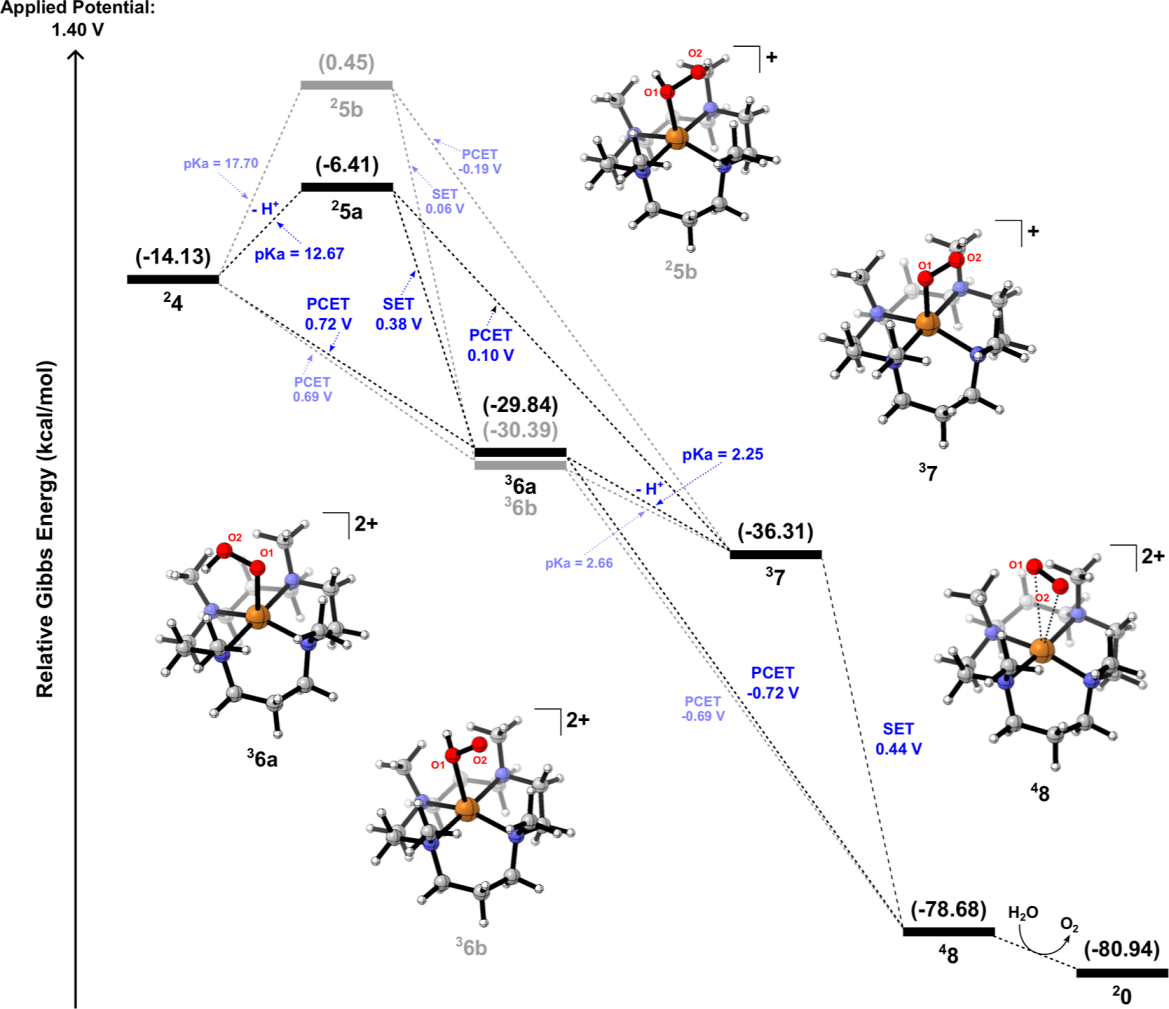
Gibbs free energy (298.15 K) diagram for SET and PCET involving intermediates ^**2**^ **4** and ^**2**^ **5a** in a descendant electrochemical step.

Figure 6 summarizes the two distinct pathways in the electrocatalytic cycle based on our computational results. The first step for both pathways comprises a proton-coupled electron transfer (PCET) followed by a single-electron transfer (SET) (black pathway) or a second PCET (gray pathway). It is important to emphasize that these electrochemical reactions are aligned with previous experimental and theoretical studies, which state that an electrochemical activation (oxidation) of the catalyst complex is fundamental to drive the formation of the O–O bond by providing an electrophilic site on the oxygen atoms, making them prone to undergo the SET-WNA mechanism. More specifically, previous studies have shown that electrochemical oxidative reaction steps can involve either the ligand^27,28,36,39^ or the copper.^23,24,27^ Ligands prone to oxidation are described as “non-innocent”, as they prevent the electron loss from the metal center, thus preserving the metal center oxidation state.

**Figure 6:**
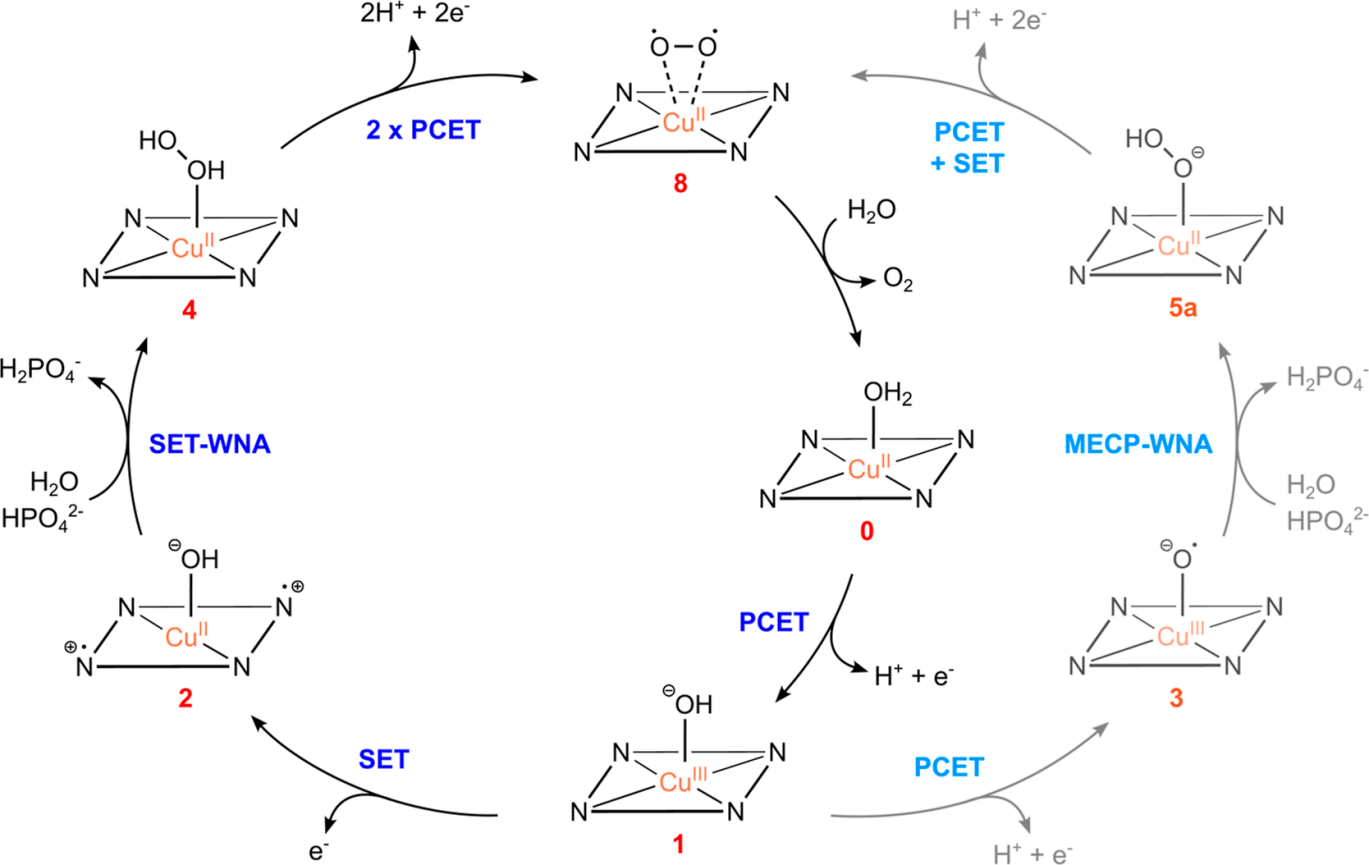
Proposed mechanism of WO catalysis with [Cu­(14-TMC)­(H_2_O)]^2+^ (**0**) as catalyst.

It is important to emphasize that these pathways differ in the O–O bond formation mechanism. The black pathway, associated with ^**4**^ **2**, proceeds via a two-step SET-WNA mechanism with doublet spin multiplicity. In contrast, the gray pathway reveals a MECP SET-WNA mechanism starting from ^**4**^ **3**, which follows a quartet state route to reach intermediate ^**4**^ **INT2**, revealing an intersystem crossing to doublet multiplicity that culminates in ^**2**^ **5a**. Since 3d transition metal complexes usually display multiconfigurational nature, theoretical treatments of WO homogeneous catalysis have revealed a potential tendency of intersystem crossings related to the oxygen–oxygen bond connection,^33,34^ in a similar fashion to what is shown in this study. Finally, upon completion of the catalytic cycle, O_2_ is released through successive PCET and SET processes.

## 4 Conclusions

The present computational work based on DFT calculations investigated electrochemical water oxidation using, as the catalyst, the single-site macrocyclic complex [Cu­(14-TMC)­(H_2_O)]_^2+^_ (**0**), with a special emphasis on scrutinizing its electrocatalytic cycle. Our results highlighted the importance of the HPO_4_^2–^ ion to facilitate the O–O bond formation compared to the pathway mediated by only water molecules, which was found to be energetically prohibitive. Furthermore, although the experimental data have been well described by a two-step SET-WNA pathway from the intermediate ^**4**^ **2** (based on the lack of pH dependence observed in the ^**3**^ **1** → ^**4**^ **2** process), our computational findings also suggest a competitive multistep SET-WNA route given by pathway ^**4**^ **3**. Finally, both ^**2**^ **4** and ^**2**^ **5a** can undergo a sequence of proton/electron losses in order to reach ^**4**^ **8** and, at this final point in the catalytic cycle, molecular oxygen is released and the catalyst is regenerated. In conclusion, our DFT calculations support a more detailed picture for this WO electrocatalytic cycle promoted by Cu­(II) complexes based on experiments previously established in the literature. For instance, the computed activation free energy showed excellent agreement with the free energy activation derived from the experimental TOF using the Eyring equation. We expect these results to motivate new experimental and computational studies on homogeneous WO catalysis based on macrocyclic Cu­(II) complexes. Additionally, new calculations are ongoing to extend these computational approaches presented here to investigate similar macrocyclic systems involving low-cost transition metals to design new promising WO macrocyclic catalysts. Furthermore, a specific and systematic computational/theoretical investigation of the self-interaction error,^61–63^ along with the description of the total magnetic state in Cu complexes using broken symmetry approaches,^76^ including benchmarking with various DFT functionals from different frameworks, represents interesting topics for future studies in this field.

## Supplementary Material



## Data Availability

The data underlying this study are available in the published article and its Supporting Information.

## Notes

Further computational data and Cartesian coordinates of all stationary points obtained with their respective SCF energies (PDF)
