# Supplementary material for: Theoretical Screening of the Water Oxidation Electrocatalytic Cycle Promoted by Single-Site Macrocyclic Copper(II) Complexes: Unraveling the Role of the HPO4 2– Anion under Neutral Conditions
Source: ACS Org Inorg Au. 2025 Jul 17;6(1):53–63. doi: 10.1021/acsorginorgau.5c00047 (PMC12879180; doi:10.1021/acsorginorgau.5c00047)
Supplement: Supplementary file 1 [file gg5c00047_si_001.pdf]

Supporting Information

Theoretical Screening of the Water Oxidation Electrocatalytic Cycle Promoted by Single-Site Macrocyclic Copper(II) Complexes: Unraveling the Role of HPO_4^{2-} anion under Neutral Conditions

João Pedro C. S. Neves[†], Joel Leitão Nascimento[a†], Bruno S. Sampaio, Roberto Rivelino, Tiago Vinicius Alves[*,†] and Vitor H. Menezes da Silva[*,†]

[†]*Departamento de Físico-Química, Instituto de Química, Universidade Federal da Bahia, Rua Barão de Jeremoabo, 147, Salvador, Bahia, 40170-115, Brazil*
[‡]*Instituto de Física, Universidade Federal da Bahia, Salvador, Bahia 40210-340, Brazil*

*E-mail address: tiagova@ufba.br, vhugomenezes@gmail.com or vhugo@iq.usp.br

Sections

S1. Benchmarking and Structural Parameters 4

S2. Spin Densities 5

S3. Analysis of intermediate ^{4}**2** 7

S4. Alternative ^{4}**3** Pathway 8

S5. Cartesian Coordinates and Electronic Energies 13

Tables

S1. Benchmarking of theoretical redox potentials against the experimental PCET value (1.64 V). All geometries were optimized at the B3LYP/def2-SVP level. Mean Unsigned Deviations (MUD) are reported relative to the experimental value. 4

S2. Selected bond lenghts from x-ray crystallography (Exp) and from DFT calculations (def2-SVP) 4

S3. Charge transfer analysis of ^{4}INT2 and ^{2}PC2 using Voronoi[1] and Hirshfeld[2] approaches. 6

Figures

S1. Optimized structure of the catalyst $[Cu(14\text{-}TMC)(H_2O)]^{2+}$; H atoms attached to carbon were omitted for clarity. 4

S2. Spin Densities of all minima and transition states found; M06L/Def2-TZVP wavefuctions were taken for this analysis. 5

S3. Spin Densities related to the MECP of Pathway^4**3**; H atoms attached to carbon were omitted for clarity. 6

S4. Spin Densities of ^{4}**2** at B3LYP-D3 and ωb97X-D optimized geometries with Def2-SVP basis set; H atoms attached to carbon were omitted for clarity. . 7

S5. Equilibrium between the η^2 and η^3 structures of ^{4}**2**. 7

S6. Gibbs free energy diagram for O–O bond formation via the concerted mechanism, considering the process from the second PCET (**1** $\rightarrow$ **3**). 8

S1. Benchmarking and Structural Parameters

Table S1.: Benchmarking of theoretical redox potentials against the experimental PCET value (1.64 V). All geometries were optimized at the B3LYP/def2-SVP level. Mean Unsigned Deviations (MUD) are reported relative to the experimental value.

Methods	PCET	MUD
B3LYP-D3/Def2-TZVP	1.96	19.3
M06L-D3/Def2-TZVP	1.49	9.08
WB97XD/Def2-TZVP	2.32	41.5
M06-2X-D3/Def2-TZVP	2.82	72.17
M06HF-D3/Def2-TZVP	4.30	162.2
M06-2X/Def2-TZVP	2.82	71.8
M06HF/Def2-TZVP	4.29	161.6

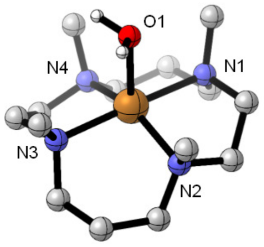

Fig. S1.: Optimized structure of the catalyst $[Cu(14\text{-}TMC)(H_2O)]^{2+}$; H atoms attached to carbon were omitted for clarity.

Table S2.: Selected bond lenghts from x-ray crystallography (Exp) and from DFT calculations (def2-SVP)

Bond Lenght	Exp.^{a}	def2-SVP
Cu(1)-O(1)	2.238(2)	2.32960
Cu(1)-N(1)	2.090(2)	2.15795
Cu(1)-N(2)	2.096(2)	2.13719
Cu(1)-N(3)	2.0968(19)	2.16725
Cu(1)-N(4)	2.090(2)	2.12073

S2. Spin Densities

Spin Densities (ρ)

^{2}**0**	^{3}**1**	^{4}**2**	^{4}**3**
ρ_{Cu} = 0.620	ρ_{Cu} = 0.865	ρ_{Cu} = 0.702	ρ_{Cu} = 0.917
ρ_{L} = 0.380	ρ_{L} = 0.758	ρ_{L} = 0.644 (N2) / 0.623 (N4) / 0.764	ρ_{L} = 0.800
ρ_{O} < 0.001	ρ_{O} = 0.377	ρ_{O} = 0.267	ρ_{O} = 1.283

^{2}**RC1**	^{2}**INT1**	^{2}**TS1**	^{2}**PC1**
ρ_{Cu} = 0.002	ρ_{Cu} = 0.670	ρ_{Cu} = 0.007	ρ_{Cu} = 0.565
ρ_{L} = 0.648 (N4) / 0.173	ρ_{L} = -0.634 (N4) / 0.064	ρ_{L} = 0.675 (N4) / 0.261	ρ_{L} = 0.434
ρ_{O1} = 0.153	ρ_{O1} = 0.738	ρ_{O1} = 0.042	ρ_{O1} = 0.001
ρ_{O2} = 0.008	ρ_{O2} = 0.209	ρ_{O2} = 0.006	ρ_{O2} < 0.001
ρ_{Phos} = 0.016	ρ_{Phos} = -0.047	ρ_{Phos} = 0.009	ρ_{Phos} < 0.001

^{4}**RC2**	^{4}**TS2**	^{4}**INT2**	^{2}**PC2**	^{2}**TS0**
ρ_{Cu} = 0.682	ρ_{Cu} = 0.675	ρ_{Cu} = 0.647	ρ_{Cu} = 0.578	ρ_{Cu} = 0.566
ρ_{L} = 0.513	ρ_{L} = 0.513	ρ_{L} = 0.475	ρ_{L} = 0.401	ρ_{L} = 0.433
ρ_{O1} = 1.336	ρ_{O1} = 1.434	ρ_{O1} = 1.315	ρ_{O1} = 0.018	ρ_{O1} = 0.001
ρ_{O2} = 0.017	ρ_{O2} = 0.350	ρ_{O2} = 0.566	ρ_{O2} = 0.002	ρ_{O2} < 0.001
ρ_{Phos} = 0.452	ρ_{Phos} = 0.010	ρ_{Phos} = -0.003	ρ_{Phos} = 0.001	

^{2}**4**	^{2}**5a**	^{2}**5b**	^{2}**6a**	^{2}**6b**
ρ_{Cu} = 0.566	ρ_{Cu} = 0.574	ρ_{Cu} = 0.563	ρ_{Cu} = 0.580	ρ_{Cu} = 0.584
ρ_{L} = 0.433	ρ_{L} = 0.296	ρ_{L} = 0.396	ρ_{L} = 0.447	ρ_{L} = 0.449
ρ_{O1} < 0.001	ρ_{O1} = 0.105	ρ_{O1} = 0.019	ρ_{O1} = 0.644	ρ_{O1} = 0.307
ρ_{O2} < 0.001	ρ_{O2} = 0.025	ρ_{O2} = 0.022	ρ_{O2} = 0.329	ρ_{O2} = 0.660

^{3}**7**		^{4}**8**	
ρ_{Cu} = 0.518	ρ_{O1} = 0.557	ρ_{Cu} = 0.559	ρ_{O1} = 0.992
ρ_{L} = 0.331	ρ_{O2} = 0.594	ρ_{L} = 0.452	ρ_{O2} = 0.997

Fig. S2.: Spin Densities of all minima and transition states found; M06L/Def2-TZVP wavefuctions were taken for this analysis.

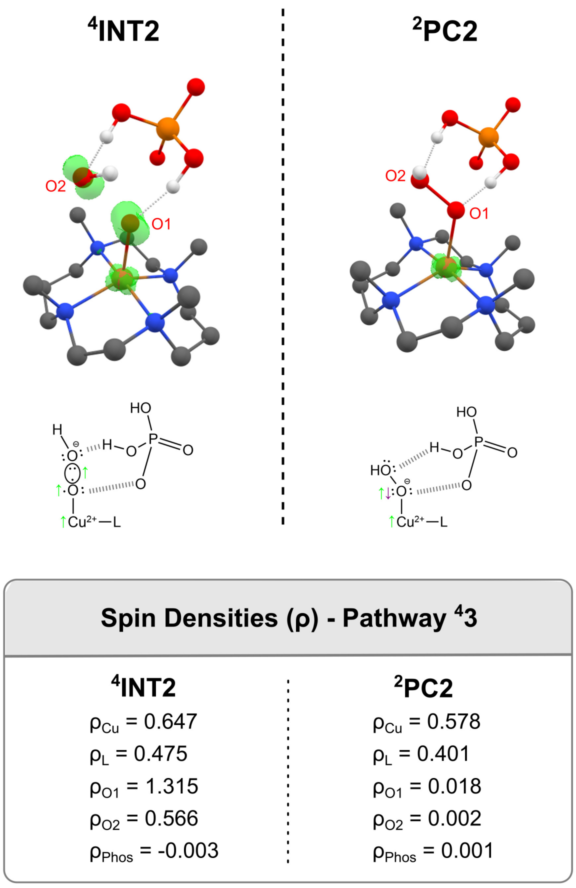

Fig. S3.: Spin Densities related to the MECP of Pathway^4**3**; H atoms attached to carbon were omitted for clarity.

Table S3.: Charge transfer analysis of ^{4}INT2 and ^{2}PC2 using Voronoi[1] and Hirshfeld[2] approaches.

System	Atom	Voronoi	Hirshfeld
^{4}INT2	O1	-0.196	-0.139
	O2	-0.353	-0.344
^{2}PC2	O1	-0.323	-0.258
	O2	-0.168	-0.134

[1] Fonseca Guerra, C.; Handgraaf, J.-W.; Baerends, E. J.; Bickelhaupt, F. M. Voronoi deformation density (VDD) charges: Assessment of the Mulliken, Bader, Hirshfeld, Weinhold, and VDD methods for charge analysis. J. Comput. Chem. 2004, 25, 189–210 DOI: 10.1002/jcc.10351

[2] Hirshfeld, F. L. Bonded-Atom Fragments for Describing Molecular Charge Densities. Theor. Chim. Acta 1977, 44 (2), 129–138, DOI: 10.1007/BF00549096

S3. Analysis of intermediate ^{4}**2**

In order to test our level of theory relative to this specific intermediate, ^{4}**2** was optimized in ωb97X-D with the same B3LYP-D3 initial guess, thus leading to the same η^2-(14-TMC) structure. Another possible structure (η^3) was also optimized, which was found to be more unstable for both functionals. These results are summarized in Figures S4 and S5 below. The present methodology (M06L-D3/Def2-TZVP//B3LYP-D3/Def2-SVP) also follow the same stability order.

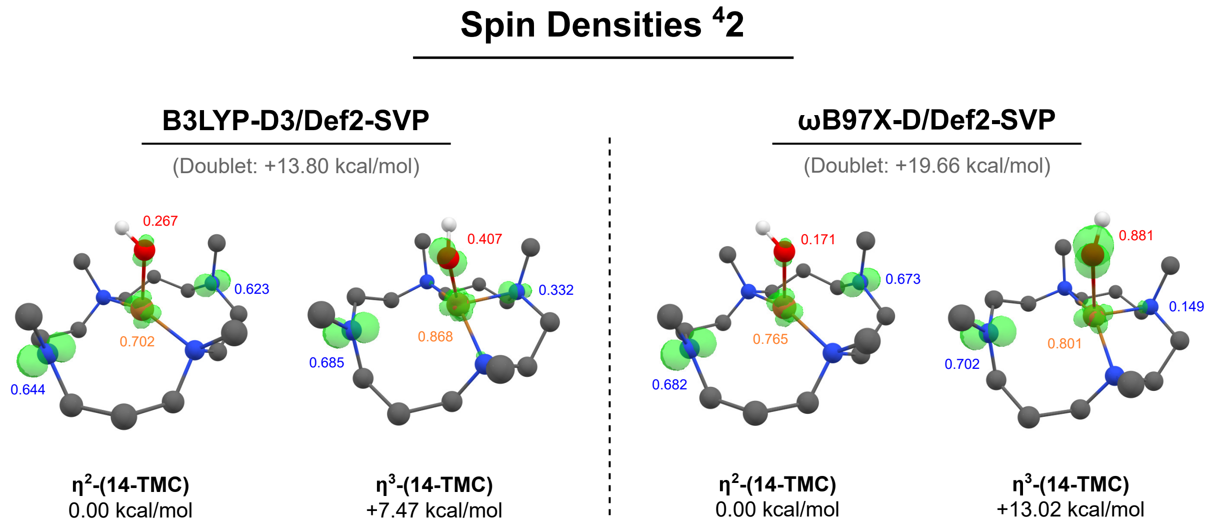

Fig. S4.: Spin Densities of ^{4}**2** at B3LYP-D3 and ωb97X-D optimized geometries with Def2-SVP basis set; H atoms attached to carbon were omitted for clarity.

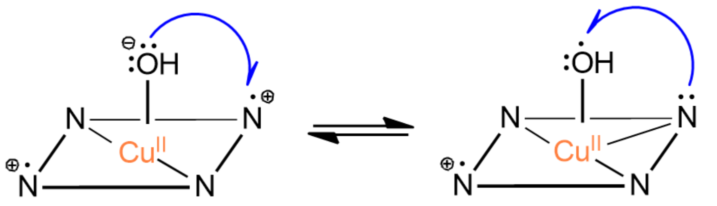

Fig. S5.: Equilibrium between the η^2 and η^3 structures of ^{4}**2**.

S4. Alternative ^{4}3 Pathway

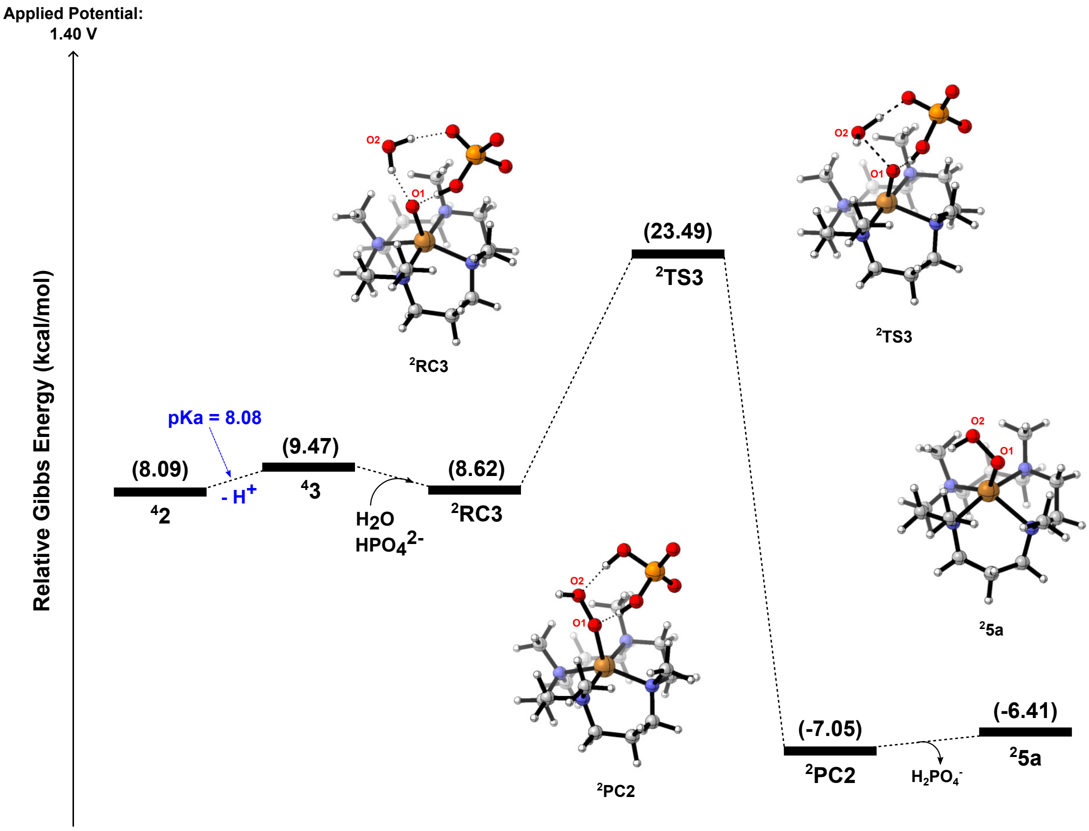

Fig. S6.: Gibbs free energy diagram for O–O bond formation via the concerted mechanism, considering the process from the second PCET (**1** → **3**).

^{2}RC3

E(UB3LYP) = -1761.59781220

29	0.036627000	-0.606441000	0.489165000
6	-2.534451000	-2.140542000	1.357687000
1	-1.905525000	-3.044156000	1.370797000
6	-3.104436000	-1.960358000	-0.042600000
6	-2.071753000	-2.006909000	-1.162268000
1	-2.595252000	-2.094985000	-2.132178000
1	-1.466098000	-2.915086000	-1.037442000
6	-1.866136000	0.387594000	-1.593989000
1	-2.388434000	0.279238000	-2.561866000
1	-2.601880000	0.629400000	-0.823969000
1	-1.166851000	1.225220000	-1.657618000
6	-0.144066000	-1.114075000	-2.338158000

6	0.928054000	-2.073068000	-1.868609000
1	0.490096000	-3.044169000	-1.603076000
6	2.571249000	-0.488665000	-1.082447000
1	2.067238000	0.267598000	-1.690399000
1	2.991135000	0.023999000	-0.213183000
1	3.398941000	-0.928578000	-1.668892000
6	2.296900000	-2.638937000	0.044576000
1	1.545410000	-3.423838000	0.220937000
1	3.070062000	-3.068768000	-0.621016000
6	2.928941000	-2.252124000	1.374248000
1	3.479224000	-3.137118000	1.731432000
1	3.689085000	-1.467539000	1.245377000
6	1.936471000	-1.894908000	2.473266000
1	2.469600000	-1.835229000	3.440215000
1	1.204134000	-2.709885000	2.557005000
6	2.072450000	0.544110000	2.381540000
1	2.561285000	0.586539000	3.371305000
1	2.847784000	0.502890000	1.611446000
1	1.485462000	1.459703000	2.231138000
6	0.158224000	-0.513352000	3.375948000
1	0.612077000	-0.774575000	4.347949000
1	-0.144690000	0.539566000	3.435026000
6	-1.045632000	-1.392317000	3.109534000
1	-1.756151000	-1.317125000	3.953572000
1	-0.741719000	-2.445290000	3.041492000
7	-1.698619000	-1.019970000	1.837375000
7	-1.138085000	-0.850815000	-1.263523000
7	1.616314000	-1.537048000	-0.672694000
7	1.176843000	-0.626784000	2.296658000
8	0.005359000	1.449825000	0.555122000
1	-3.787323000	-2.806576000	-0.219671000
1	-3.731478000	-1.058819000	-0.106812000
1	-3.360983000	-2.308640000	2.075854000
1	0.298726000	-0.150695000	-2.623953000
1	-0.650356000	-1.516023000	-3.233246000

1	1.651917000	-2.257652000	-2.682731000
6	-2.499127000	0.202516000	2.023560000
1	-2.871063000	0.581024000	1.067602000
1	-3.359957000	0.006740000	2.690184000
1	-1.883031000	0.994209000	2.462291000
8	2.020413000	4.146668000	-2.952170000
15	1.036423000	3.103197000	-2.328530000
8	-0.425231000	3.524921000	-2.234223000
8	1.611911000	2.706895000	-0.887234000
8	1.316102000	1.949267000	-3.356300000
1	0.927100000	2.174501000	-0.281645000
8	-1.927709000	3.225402000	0.060244000
1	-1.449622000	3.416678000	-0.778718000
1	-1.291966000	2.599954000	0.478122000

^{2}TS3

E(UB3LYP) = -1761.56596312

29	53.709102000	2.805054000	4.460570000
6	51.323951000	1.053485000	5.380430000
1	51.997348000	0.188134000	5.469205000
6	50.779986000	1.115053000	3.959823000
6	51.846896000	1.208889000	2.879358000
1	51.383321000	1.009181000	1.897355000
1	52.598155000	0.426501000	3.053217000
6	51.668816000	3.633747000	2.445661000
1	51.122098000	3.414795000	1.512642000
1	50.945004000	3.794399000	3.247255000
1	52.257045000	4.547829000	2.324091000
6	53.578053000	2.415315000	1.646395000
6	54.762396000	1.556612000	2.022578000
1	54.448086000	0.534882000	2.270366000
6	56.359724000	3.218502000	2.762286000
1	55.831595000	3.915390000	2.104250000
1	56.732393000	3.785743000	3.618874000
1	57.222802000	2.793450000	2.217484000

6	56.189729000	1.077796000	3.931290000
1	55.479876000	0.256463000	4.113679000
1	56.994826000	0.681622000	3.282839000
6	56.777509000	1.530913000	5.259894000
1	57.400941000	0.702432000	5.631058000
1	57.462074000	2.382024000	5.134551000
6	55.746065000	1.789162000	6.348062000
1	56.261035000	1.894039000	7.319305000
1	55.090909000	0.911163000	6.419307000
6	55.653953000	4.240739000	6.257779000
1	56.190626000	4.288053000	7.220758000
1	56.380177000	4.283237000	5.443013000
1	54.978404000	5.095535000	6.164657000
6	53.871815000	3.003956000	7.309515000
1	54.387307000	2.814962000	8.265791000
1	53.447120000	4.013734000	7.361926000
6	52.789391000	1.977726000	7.081954000
1	52.067433000	2.001469000	7.916711000
1	53.205373000	0.963659000	7.042459000
7	52.096946000	2.242753000	5.798911000
7	52.575430000	2.505983000	2.750680000
7	55.463838000	2.128581000	3.191924000
7	54.870136000	2.991336000	6.199964000
8	54.049586000	4.585646000	3.922986000
1	50.249465000	0.165815000	3.785936000
1	50.018882000	1.900021000	3.845376000
1	50.492962000	0.902739000	6.094166000
1	53.899159000	3.437131000	1.412828000
1	53.081001000	2.007397000	0.750100000
1	55.448754000	1.481317000	1.160802000
6	51.244184000	3.447239000	5.929958000
1	50.539862000	3.514488000	5.097645000
1	50.661428000	3.398064000	6.865470000
1	51.854611000	4.358068000	5.924839000
8	55.233696000	8.101271000	1.005497000

15	54.639773000	6.815380000	1.583870000
8	53.186096000	7.003404000	2.160339000
8	55.584705000	6.497303000	2.937756000
8	54.727598000	5.576156000	0.665784000
1	55.169692000	5.735649000	3.412237000
8	52.905057000	6.322083000	4.545368000
1	53.015695000	6.622250000	3.536914000
1	53.715014000	6.652808000	4.963950000

S5. Cartesian Coordinates and Electronic Energies

^{2}0

E(UB3LYP) = -1043.62402508 hartree

29	4.206701000	2.820079000	2.815955000
6	1.792509000	1.189118000	3.615500000
1	2.453638000	0.309930000	3.602419000
6	1.182399000	1.362042000	2.232575000
6	2.192687000	1.418510000	1.093400000
1	1.662400000	1.309314000	0.129482000
1	2.877376000	0.563922000	1.187656000
6	2.177832000	3.829853000	0.721905000
1	1.626439000	3.670871000	-0.222132000
1	1.454030000	4.017151000	1.520656000
1	2.812128000	4.715892000	0.626764000
6	4.023029000	2.519306000	-0.068141000
6	5.205458000	1.684217000	0.364993000
1	4.883345000	0.664735000	0.614246000
6	6.644512000	3.451432000	1.201199000
1	6.008747000	4.183300000	0.690081000
1	7.058642000	3.935610000	2.090067000
1	7.476894000	3.169860000	0.531535000
6	6.697263000	1.218764000	2.203480000
1	6.063376000	0.328624000	2.333347000
1	7.501927000	0.948768000	1.494371000
6	7.304949000	1.583219000	3.549950000
1	7.967983000	0.748081000	3.827048000
1	7.959433000	2.464097000	3.474322000
6	6.300452000	1.726751000	4.685884000
1	6.841390000	1.765238000	5.649299000
1	5.661843000	0.832228000	4.706250000
6	6.179689000	4.160056000	4.780911000
1	6.732028000	4.144475000	5.737712000
1	6.901106000	4.287453000	3.968323000

1	5.499574000	5.019269000	4.786299000
6	4.408253000	2.846149000	5.720263000
1	4.894333000	2.520774000	6.656350000
1	4.031053000	3.861725000	5.892857000
6	3.269503000	1.914346000	5.376706000
1	2.538848000	1.889451000	6.204459000
1	3.640821000	0.890666000	5.237582000
7	2.604815000	2.328569000	4.115913000
7	3.019648000	2.653700000	1.021522000
7	5.851107000	2.262529000	1.569897000
7	5.405520000	2.914642000	4.618971000
8	4.218584000	5.131022000	2.521966000
1	3.786108000	5.495999000	3.309635000
1	5.135965000	5.435829000	2.592691000
1	0.560356000	0.470587000	2.054434000
1	0.487484000	2.214164000	2.200220000
1	0.992639000	0.986271000	4.351786000
1	4.351289000	3.529179000	-0.343849000
1	3.555105000	2.076628000	-0.964695000
1	5.934084000	1.603222000	-0.460987000
6	1.769923000	3.523504000	4.349826000
1	1.314055000	3.865084000	3.416885000
1	0.965585000	3.298921000	5.073046000
1	2.382050000	4.343213000	4.743518000

^{3}**1**

E(UB3LYP) = -1042.93488173 hartree

29	4.218100000	2.815607000	2.838811000
6	1.917108000	1.174650000	3.598219000
1	2.664807000	0.369998000	3.529257000
6	1.242319000	1.343056000	2.243486000
6	2.195088000	1.450587000	1.058765000
1	1.621212000	1.367451000	0.117578000
1	2.891836000	0.602117000	1.090001000

6	2.158962000	3.876974000	0.731940000
1	1.646364000	3.761559000	-0.239740000
1	1.402981000	4.007032000	1.511487000
1	2.787853000	4.772076000	0.711662000
6	4.025607000	2.604488000	-0.081141000
6	5.195348000	1.749393000	0.340142000
1	4.871116000	0.723093000	0.551994000
6	6.690093000	3.447504000	1.237737000
1	6.106639000	4.185232000	0.674197000
1	7.059679000	3.932341000	2.144580000
1	7.550176000	3.125323000	0.626051000
6	6.592880000	1.189094000	2.228214000
1	5.875623000	0.369050000	2.385214000
1	7.351973000	0.829597000	1.509688000
6	7.268420000	1.520832000	3.551504000
1	7.888969000	0.646621000	3.804085000
1	7.971398000	2.360034000	3.447882000
6	6.318683000	1.718985000	4.726224000
1	6.898149000	1.764089000	5.666539000
1	5.654839000	0.845924000	4.792766000
6	6.257124000	4.166433000	4.792589000
1	6.717366000	4.208587000	5.795658000
1	7.055669000	4.201112000	4.045564000
1	5.601985000	5.032185000	4.653562000
6	4.436508000	2.915872000	5.737908000
1	4.902597000	2.623065000	6.694639000
1	4.065783000	3.941381000	5.857090000
6	3.297786000	1.977920000	5.414626000
1	2.563792000	1.979589000	6.237984000
1	3.660901000	0.948629000	5.304234000
7	2.625760000	2.363589000	4.144766000
7	2.998946000	2.695775000	0.986768000
7	5.839806000	2.287889000	1.566989000
7	5.464519000	2.931345000	4.665870000
8	4.037276000	4.749687000	2.842340000

1	4.883788000	5.093064000	2.507271000
1	0.654829000	0.426007000	2.080390000
1	0.508383000	2.161850000	2.253945000
1	1.165392000	0.864593000	4.346673000
1	4.358237000	3.624828000	-0.308002000
1	3.573180000	2.194782000	-1.000910000
1	5.936516000	1.695016000	-0.475141000
6	1.728284000	3.511940000	4.362253000
1	1.301402000	3.854527000	3.417125000
1	0.908745000	3.224066000	5.042750000
1	2.290478000	4.343139000	4.800254000

$^{4}\mathbf{2}$

E(UB3LYP) = -1042.72439761 hartree

29	4.232279000	2.783486000	2.830452000
6	1.822422000	1.464615000	3.514248000
1	2.338906000	0.517268000	3.302290000
6	1.169004000	1.996487000	2.228974000
6	1.796235000	1.573012000	0.882783000
1	0.973590000	1.503960000	0.143272000
1	2.248037000	0.574582000	0.945681000
6	2.505281000	3.887792000	0.227679000
1	2.969654000	4.334183000	-0.660963000
1	1.427272000	4.093009000	0.259260000
1	2.980356000	4.343832000	1.129841000
6	3.947004000	1.929323000	-0.384987000
6	5.013918000	1.342479000	0.554761000
1	4.574768000	0.535577000	1.158332000
6	6.286091000	3.438355000	0.792225000
1	5.512384000	4.059912000	0.325640000
1	6.836581000	4.061026000	1.507354000
1	6.984404000	3.077841000	0.018193000
6	6.703322000	1.524771000	2.277609000
1	6.236914000	0.574355000	2.572746000

1	7.517731000	1.281610000	1.574538000
6	7.310609000	2.205175000	3.517629000
1	8.338666000	1.824411000	3.590618000
1	7.414432000	3.289166000	3.373804000
6	6.671364000	1.883246000	4.889109000
1	7.482969000	1.890962000	5.643192000
1	6.243311000	0.872558000	4.899483000
6	5.898252000	4.221539000	5.364702000
1	5.467148000	4.714683000	6.246507000
1	6.966139000	4.451035000	5.258929000
1	5.353543000	4.602218000	4.458218000
6	4.512786000	2.282660000	6.125860000
1	4.880324000	1.537676000	6.852750000
1	4.049877000	3.100852000	6.687333000
6	3.489164000	1.548184000	5.251856000
1	2.732541000	1.143359000	5.946661000
1	3.965473000	0.695722000	4.748436000
7	2.809568000	2.359721000	4.203318000
7	2.751481000	2.473261000	0.253937000
7	5.672973000	2.296174000	1.498133000
7	5.684179000	2.809078000	5.432338000
8	4.196840000	4.688679000	2.853390000
1	4.873069000	5.044456000	2.256941000
1	0.155212000	1.573516000	2.210040000
1	1.023540000	3.084356000	2.270632000
1	1.025838000	1.243662000	4.245218000
1	4.367429000	2.700258000	-1.039521000
1	3.612257000	1.089112000	-1.017628000
1	5.778744000	0.893297000	-0.102004000
6	2.150140000	3.556238000	4.760698000
1	1.600500000	4.074737000	3.967716000
1	1.449315000	3.268431000	5.562616000
1	2.897441000	4.253682000	5.153494000

^{4}**3**

E(UB3LYP) = -1042.25779341 hartree

29	4.218289000	2.741453000	2.836684000
6	1.894852000	1.154495000	3.583225000
1	2.634457000	0.344637000	3.489295000
6	1.206346000	1.365195000	2.241394000
6	2.145190000	1.473187000	1.046133000
1	1.555274000	1.442615000	0.111963000
1	2.811533000	0.599798000	1.041061000
6	2.215107000	3.912394000	0.763695000
1	1.717566000	3.856080000	-0.220604000
1	1.450305000	4.046814000	1.533470000
1	2.884039000	4.782630000	0.773917000
6	4.023334000	2.560970000	-0.080167000
6	5.186642000	1.706523000	0.361984000
1	4.849266000	0.691265000	0.607303000
6	6.660567000	3.447183000	1.223666000
1	6.054654000	4.175420000	0.674477000
1	7.039375000	3.941645000	2.120997000
1	7.512979000	3.134698000	0.596509000
6	6.623831000	1.198101000	2.238230000
1	5.927645000	0.362385000	2.408658000
1	7.388942000	0.850797000	1.520636000
6	7.293014000	1.566491000	3.555443000
1	7.940230000	0.715182000	3.818776000
1	7.969691000	2.425275000	3.438731000
6	6.342285000	1.750683000	4.731812000
1	6.924614000	1.835365000	5.667303000
1	5.710200000	0.855941000	4.818782000
6	6.178741000	4.199876000	4.762731000
1	6.632214000	4.286754000	5.766011000
1	6.976795000	4.250471000	4.016831000
1	5.488881000	5.040048000	4.609966000
6	4.419354000	2.875347000	5.744272000
1	4.898385000	2.567610000	6.689194000

1	4.034983000	3.891942000	5.890797000
6	3.294414000	1.926125000	5.403985000
1	2.559316000	1.902571000	6.226156000
1	3.672733000	0.904295000	5.273282000
7	2.620377000	2.327691000	4.141126000
7	3.000338000	2.685939000	0.991257000
7	5.839413000	2.272906000	1.572776000
7	5.439021000	2.928832000	4.663536000
8	4.134497000	4.653504000	2.744760000
1	0.594523000	0.466772000	2.065347000
1	0.492883000	2.201136000	2.278898000
1	1.148537000	0.838018000	4.334204000
1	4.362501000	3.571264000	-0.338077000
1	3.561927000	2.130298000	-0.984914000
1	5.924764000	1.619421000	-0.453243000
6	1.742354000	3.489829000	4.369205000
1	1.309939000	3.841394000	3.430163000
1	0.926298000	3.212500000	5.058042000
1	2.320129000	4.314163000	4.799342000

^{2}RC1

E(UB3LYP) = -1762.07029652 hartree

29	-0.433482000	-0.637922000	-0.983475000
6	-2.767403000	-2.051630000	-0.080140000
1	-2.193386000	-2.959484000	-0.314991000
6	-3.572056000	-1.641174000	-1.298815000
6	-2.767380000	-1.661546000	-2.586348000
1	-3.427946000	-1.474538000	-3.450585000
1	-2.314812000	-2.655225000	-2.719664000
6	-2.172966000	0.724091000	-2.698210000
1	-2.692062000	0.888589000	-3.657110000
1	-2.870381000	0.920961000	-1.878988000
1	-1.326065000	1.419728000	-2.619238000
6	-0.774137000	-0.938313000	-3.811938000

6	0.403260000	-1.751536000	-3.354167000
1	0.061112000	-2.715986000	-2.954816000
6	1.727192000	0.232332000	-2.689379000
1	0.952688000	0.975942000	-2.907327000
1	2.369069000	0.627828000	-1.899969000
1	2.331363000	0.051425000	-3.593685000
6	2.147144000	-1.966835000	-1.679699000
1	1.716906000	-2.977029000	-1.700197000
1	2.980177000	-1.958074000	-2.403719000
6	2.706260000	-1.686784000	-0.283917000
1	3.651174000	-2.245306000	-0.230029000
1	2.965076000	-0.630739000	-0.162940000
6	1.783293000	-2.178807000	0.855541000
1	2.303824000	-2.946600000	1.456584000
1	0.896034000	-2.658873000	0.430944000
6	2.363028000	-0.383436000	2.480482000
1	3.187491000	-1.047365000	2.786350000
1	2.759304000	0.384006000	1.800242000
1	1.926448000	0.119685000	3.349805000
6	0.013781000	-1.146112000	2.384434000
1	0.094142000	-1.711856000	3.333333000
1	-0.200183000	-0.107767000	2.678316000
6	-1.116111000	-1.794411000	1.612545000
1	-1.909048000	-1.983826000	2.354656000
1	-0.820474000	-2.777185000	1.230651000
7	-1.790719000	-1.061868000	0.482441000
7	-1.669962000	-0.668793000	-2.636615000
7	1.117074000	-1.041642000	-2.250192000
7	1.349980000	-1.163231000	1.799255000
8	0.681419000	0.282092000	0.104416000
1	-4.364257000	-2.397139000	-1.411744000
1	-4.091657000	-0.684356000	-1.147461000
1	-3.458524000	-2.301324000	0.742475000
1	-0.455920000	0.021363000	-4.234200000
1	-1.335531000	-1.469713000	-4.594195000

1	1.108241000	-1.949968000	-4.178769000
6	-2.459436000	0.165104000	0.969309000
1	-2.902465000	0.710252000	0.130689000
1	-3.252813000	-0.112893000	1.682836000
1	-1.732399000	0.824812000	1.450148000
8	-0.027230000	1.916917000	3.555839000
15	0.427543000	2.824775000	2.406561000
8	1.952420000	3.057032000	2.293615000
8	-0.246806000	4.309299000	2.770990000
8	-0.205381000	2.426931000	1.024793000
1	0.197059000	4.997322000	2.250954000
8	2.908877000	1.906675000	0.091022000
1	2.611501000	2.407772000	0.903258000
1	2.136132000	1.319888000	-0.051510000
1	0.187183000	1.090573000	0.534773000

^{2}INT1

E(UB3LYP) = -1762.06317314 hartree

29	5.441664000	1.987115000	0.521506000
6	3.169405000	0.386208000	1.514881000
1	3.812691000	-0.496991000	1.385406000
6	2.413110000	0.632764000	0.217975000
6	3.269832000	0.557507000	-1.037830000
1	2.619710000	0.532210000	-1.931478000
1	3.843986000	-0.380950000	-1.022709000
6	3.568246000	2.953033000	-1.472627000
1	3.007086000	2.908538000	-2.422803000
1	2.872241000	3.196108000	-0.662724000
1	4.321711000	3.749137000	-1.532422000
6	5.192759000	1.342211000	-2.312553000
6	6.440824000	0.690013000	-1.758784000
1	6.177637000	-0.258569000	-1.268435000
6	7.558104000	2.833888000	-1.310000000
1	6.715677000	3.455461000	-1.635339000

1	8.101611000	3.393119000	-0.542884000
1	8.220296000	2.634768000	-2.170307000
6	8.199868000	0.845269000	-0.064141000
1	8.026640000	-0.236833000	-0.150825000
1	9.123593000	1.061858000	-0.624561000
6	8.377850000	1.228637000	1.405228000
1	9.433330000	1.148102000	1.698097000
1	8.072811000	2.272661000	1.553381000
6	7.523456000	0.304935000	2.315678000
1	8.076628000	-0.620002000	2.536460000
1	6.587074000	0.031447000	1.810279000
6	8.258772000	1.264603000	4.489539000
1	7.900995000	1.116388000	5.520856000
1	9.146408000	0.655737000	4.285311000
1	8.476804000	2.353927000	4.387507000
6	5.976315000	1.714706000	3.683324000
1	5.899408000	2.075596000	4.714120000
1	6.132584000	2.613100000	3.061407000
6	4.716484000	0.970568000	3.235866000
1	3.982301000	1.008131000	4.057018000
1	4.942251000	-0.090093000	3.077214000
7	4.052227000	1.487822000	2.003971000
7	4.241847000	1.664395000	-1.208379000
7	7.071467000	1.559354000	-0.732574000
7	7.204412000	0.957622000	3.563822000
8	5.930281000	3.866516000	1.136208000
1	1.672051000	-0.177975000	0.136081000
1	1.828749000	1.563898000	0.257077000
1	2.449627000	0.155614000	2.320658000
1	5.441061000	2.269313000	-2.842724000
1	4.712260000	0.676561000	-3.047046000
1	7.156005000	0.470332000	-2.571024000
6	3.275590000	2.706828000	2.331789000
1	2.879888000	3.159551000	1.416544000
1	2.437407000	2.457480000	3.005579000

1	3.929252000	3.438253000	2.821067000
8	6.283576000	4.276421000	5.817460000
15	6.716378000	4.909251000	4.501235000
8	8.048588000	4.337332000	3.916303000
8	7.016739000	6.503566000	4.877803000
8	5.616067000	4.948895000	3.388058000
1	7.213531000	7.001184000	4.068313000
8	8.130699000	4.623339000	1.381955000
1	8.118648000	4.515605000	2.401844000
1	7.665769000	5.460650000	1.232200000
1	5.837587000	4.295925000	2.089828000

^{2}TS1

E(UB3LYP) = -1762.03619397 hartree

29	5.648323000	2.057893000	0.481242000
6	3.400919000	0.433211000	1.322199000
1	4.042116000	-0.432334000	1.095958000
6	2.627730000	0.819337000	0.067298000
6	3.447893000	0.831724000	-1.215911000
1	2.773384000	0.931965000	-2.087982000
1	3.957259000	-0.139025000	-1.311567000
6	3.889058000	3.223124000	-1.456740000
1	3.342265000	3.303561000	-2.415135000
1	3.190099000	3.437299000	-0.639676000
1	4.685177000	3.980431000	-1.432553000
6	5.432553000	1.592323000	-2.393199000
6	6.599206000	0.752975000	-1.902338000
1	6.226600000	-0.206187000	-1.514707000
6	7.993380000	2.654068000	-1.265676000
1	7.249821000	3.397292000	-1.579756000
1	8.562608000	3.101754000	-0.446072000
1	8.671589000	2.442397000	-2.113932000
6	8.319452000	0.497573000	-0.204023000
1	7.893365000	-0.516779000	-0.219300000

1	9.217328000	0.469799000	-0.849167000
6	8.739128000	0.853391000	1.224206000
1	9.706407000	0.375905000	1.432375000
1	8.892518000	1.934750000	1.303734000
6	7.692659000	0.376863000	2.256203000
1	8.065054000	-0.529790000	2.772221000
1	6.763967000	0.087756000	1.754479000
6	8.433654000	2.092708000	3.905065000
1	9.340038000	1.474853000	3.976572000
1	8.646830000	2.964327000	3.265854000
1	8.118209000	2.465702000	4.886184000
6	6.030225000	1.513770000	3.842041000
1	6.077316000	1.132489000	4.879505000
1	5.867809000	2.596230000	3.952902000
6	4.872383000	0.822314000	3.151398000
1	4.072267000	0.741401000	3.908326000
1	5.142410000	-0.208397000	2.891482000
7	4.282113000	1.472156000	1.941952000
7	4.482670000	1.885245000	-1.289543000
7	7.329921000	1.422811000	-0.804751000
7	7.368900000	1.330341000	3.296459000
8	6.793147000	3.233132000	1.465350000
1	1.855714000	0.044433000	-0.064131000
1	2.076822000	1.761071000	0.206804000
1	2.681929000	0.114817000	2.099400000
1	5.793203000	2.543707000	-2.803689000
1	4.918284000	1.070873000	-3.218836000
1	7.279062000	0.529445000	-2.746614000
6	3.504011000	2.666673000	2.337167000
1	3.154744000	3.198965000	1.445572000
1	2.631995000	2.374530000	2.950068000
1	4.140121000	3.353190000	2.904439000
8	6.648724000	4.469360000	5.676158000
15	6.593484000	5.293596000	4.409325000
8	8.078120000	5.521734000	3.796517000

8	6.135195000	6.801392000	4.871622000
8	5.678567000	4.827597000	3.262438000
1	6.020732000	7.383309000	4.102267000
8	8.166292000	4.528839000	1.417807000
1	8.113662000	5.186677000	2.835934000
1	7.703615000	5.068367000	0.754883000
1	6.314507000	3.785055000	2.161789000

^{2}PC1

E(UB3LYP) = -1762.11835547 hartree

29	-0.106687000	-0.782092000	-0.674187000
6	-2.693438000	-2.134705000	-0.126881000
1	-2.132866000	-3.021339000	-0.460887000
6	-3.366221000	-1.492338000	-1.331356000
6	-2.442446000	-1.251216000	-2.517431000
1	-3.040925000	-0.953904000	-3.398213000
1	-1.946338000	-2.199014000	-2.767345000
6	-1.983199000	1.121234000	-2.207105000
1	-2.528920000	1.370060000	-3.135023000
1	-2.686755000	1.171774000	-1.371514000
1	-1.194638000	1.862703000	-2.046912000
6	-0.454698000	-0.230175000	-3.479521000
6	0.521499000	-1.384138000	-3.405175000
1	-0.012415000	-2.343156000	-3.409610000
6	2.406212000	-0.321395000	-2.322743000
1	1.994935000	0.624466000	-2.688136000
1	2.900580000	-0.111695000	-1.371704000
1	3.154001000	-0.692322000	-3.046213000
6	1.867995000	-2.661747000	-1.852841000
1	1.013894000	-3.356187000	-1.841872000
1	2.528793000	-2.965270000	-2.686069000
6	2.620471000	-2.784791000	-0.534952000
1	3.069709000	-3.790406000	-0.522140000
1	3.467354000	-2.084110000	-0.489380000

6	1.746101000	-2.691464000	0.709772000
1	2.337939000	-2.980193000	1.597995000
1	0.926657000	-3.417499000	0.614613000
6	2.177875000	-0.370783000	1.322877000
1	2.705143000	-0.681585000	2.242689000
1	2.913511000	-0.277463000	0.520124000
1	1.719534000	0.607358000	1.491605000
6	0.196250000	-1.455518000	2.125423000
1	0.650341000	-2.043742000	2.941655000
1	0.035048000	-0.439168000	2.506822000
6	-1.115469000	-2.071978000	1.699400000
1	-1.801039000	-2.145345000	2.561214000
1	-0.958789000	-3.091264000	1.323495000
7	-1.736622000	-1.271094000	0.613903000
7	-1.383723000	-0.224054000	-2.316877000
7	1.324672000	-1.312319000	-2.157751000
7	1.138935000	-1.360077000	0.978481000
8	0.654792000	1.646203000	-0.772428000
1	-4.135035000	-2.201450000	-1.677301000
1	-3.911488000	-0.580021000	-1.047939000
1	-3.464989000	-2.480796000	0.585911000
1	0.087956000	0.723416000	-3.474459000
1	-1.027092000	-0.275736000	-4.422318000
1	1.183718000	-1.377765000	-4.288402000
6	-2.409006000	-0.091008000	1.192247000
1	-2.718287000	0.607640000	0.411623000
1	-3.300153000	-0.404250000	1.765478000
1	-1.729395000	0.445455000	1.860293000
8	-0.517366000	1.953741000	3.412018000
15	-0.212521000	2.959940000	2.326975000
8	1.407319000	3.091489000	2.131800000
8	-0.627973000	4.423122000	2.928472000
8	-0.840643000	2.798074000	0.934310000
1	-0.543521000	5.116654000	2.252938000
8	1.904439000	2.276640000	-0.435255000

1	1.665513000	2.898958000	1.198392000
1	1.965559000	2.990638000	-1.096121000
1	0.008846000	2.078053000	-0.105042000

^{2}TS0

E(UB3LYP) = -1118.61103072 hartree

29	6.676083000	4.016492000	3.201275000
6	4.211775000	2.534810000	4.042735000
1	4.857891000	1.645042000	4.006004000
6	3.568820000	2.731989000	2.678294000
6	4.547320000	2.744265000	1.510991000
1	3.983431000	2.688124000	0.561946000
1	5.175855000	1.844643000	1.571141000
6	4.697681000	5.158230000	1.157432000
1	4.146793000	5.064391000	0.204589000
1	3.977497000	5.369996000	1.953178000
1	5.394561000	5.999366000	1.088443000
6	6.433361000	3.717511000	0.328734000
6	7.575556000	2.829280000	0.766235000
1	7.200764000	1.837827000	1.052641000
6	9.128362000	4.534023000	1.526590000
1	8.534389000	5.271878000	0.975258000
1	9.561669000	5.038144000	2.393994000
1	9.944607000	4.175286000	0.874171000
6	9.060106000	2.336227000	2.606409000
1	8.368466000	1.499546000	2.788608000
1	9.827645000	1.980631000	1.894123000
6	9.723114000	2.720599000	3.921349000
1	10.359893000	1.869354000	4.210471000
1	10.409509000	3.570435000	3.792875000
6	8.761966000	2.946564000	5.081637000
1	9.337146000	3.026908000	6.022269000
1	8.109264000	2.066530000	5.172248000
6	8.676123000	5.386995000	5.080077000

1	9.184934000	5.433589000	6.059728000
1	9.434705000	5.430244000	4.293782000
1	8.013418000	6.251422000	4.971604000
6	6.900682000	4.133151000	6.097038000
1	7.388891000	3.809504000	7.032274000
1	6.560540000	5.164249000	6.255623000
6	5.729044000	3.232383000	5.783150000
1	5.010247000	3.235158000	6.621244000
1	6.069190000	2.197464000	5.646846000
7	5.057408000	3.657164000	4.528048000
7	5.455789000	3.921563000	1.432823000
7	8.272328000	3.407302000	1.943749000
7	7.884057000	4.145154000	4.981346000
8	7.261979000	6.488062000	2.622210000
1	2.905642000	1.867143000	2.518462000
1	2.908739000	3.611905000	2.665516000
1	3.428898000	2.341974000	4.799432000
1	6.810018000	4.702919000	0.027964000
1	5.928918000	3.282692000	-0.551129000
1	8.286000000	2.683348000	-0.066199000
6	4.253625000	4.869831000	4.772485000
1	3.801928000	5.232879000	3.844390000
1	3.441771000	4.659859000	5.491345000
1	4.886343000	5.663646000	5.187047000
8	6.220341000	7.341188000	3.460162000
1	5.438097000	6.751263000	3.464218000
1	6.477450000	7.477950000	2.403317000

^{2}**4**

E(UB3LYP) = -1118.68867465 hartree

29	6.598898000	4.121730000	3.186523000
6	4.096198000	2.666568000	4.020104000
1	4.710088000	1.753674000	4.004026000
6	3.485188000	2.867239000	2.641553000

6	4.488325000	2.868322000	1.494627000
1	3.944718000	2.795149000	0.535149000
1	5.123427000	1.975489000	1.579861000
6	4.610155000	5.280022000	1.144562000
1	4.026885000	5.161576000	0.214201000
1	3.919715000	5.508293000	1.961359000
1	5.288552000	6.130246000	1.020355000
6	6.366853000	3.866156000	0.312641000
6	7.507801000	2.968037000	0.734195000
1	7.132527000	1.971720000	1.002453000
6	9.094273000	4.632393000	1.516617000
1	8.554344000	5.338063000	0.876326000
1	9.456471000	5.184178000	2.387674000
1	9.958975000	4.239086000	0.953296000
6	8.963491000	2.439352000	2.590212000
1	8.251611000	1.621764000	2.781193000
1	9.719860000	2.058871000	1.878758000
6	9.640524000	2.823899000	3.897942000
1	10.280162000	1.973376000	4.182717000
1	10.324885000	3.674149000	3.761044000
6	8.690432000	3.049830000	5.066569000
1	9.274566000	3.142281000	6.000392000
1	8.048329000	2.163500000	5.169942000
6	8.569429000	5.491080000	5.070327000
1	9.096862000	5.535240000	6.040017000
1	9.310219000	5.558042000	4.268729000
1	7.888226000	6.344638000	4.989956000
6	6.814971000	4.201302000	6.088675000
1	7.304146000	3.854561000	7.014725000
1	6.474375000	5.227492000	6.273565000
6	5.645898000	3.307372000	5.750675000
1	4.925554000	3.286921000	6.587340000
1	5.989252000	2.277206000	5.588288000
7	4.975649000	3.761454000	4.505843000
7	5.387426000	4.054640000	1.418307000

7	8.204019000	3.526386000	1.921234000
7	7.796660000	4.237360000	4.970436000
8	7.163600000	6.649092000	2.671747000
1	2.817193000	2.008307000	2.470294000
1	2.834203000	3.753264000	2.611387000
1	3.294159000	2.510494000	4.765098000
1	6.746069000	4.857146000	0.032628000
1	5.862354000	3.450472000	-0.576458000
1	8.217502000	2.836757000	-0.101115000
6	4.215586000	5.000857000	4.762017000
1	3.670658000	5.313450000	3.866786000
1	3.486244000	4.849220000	5.577259000
1	4.900873000	5.808066000	5.048360000
8	6.187954000	7.492220000	3.303226000
1	5.419125000	6.899081000	3.382458000
1	6.993908000	6.785143000	1.721979000

^{4}RC2

E(UB3LYP) = -1761.58942909 hartree

29	-0.122943000	-0.871333000	0.419082000
6	-2.597928000	-2.333541000	1.399851000
1	-1.949240000	-3.210656000	1.545070000
6	-3.159648000	-2.373946000	-0.014835000
6	-2.116474000	-2.492502000	-1.120217000
1	-2.622265000	-2.744826000	-2.070704000
1	-1.441799000	-3.326666000	-0.879791000
6	-2.100531000	-0.151860000	-1.825109000
1	-2.664429000	-0.444478000	-2.729591000
1	-2.811988000	0.168583000	-1.059954000
1	-1.449373000	0.693283000	-2.064081000
6	-0.250146000	-1.573216000	-2.388250000
6	0.904848000	-2.357481000	-1.809130000
1	0.557171000	-3.324391000	-1.422794000
6	2.362328000	-0.510659000	-1.211416000

1	1.734047000	0.223901000	-1.726663000
1	2.868595000	0.010386000	-0.394760000
1	3.127598000	-0.897703000	-1.908001000
6	2.345727000	-2.574572000	0.112823000
1	1.689227000	-3.426592000	0.345624000
1	3.165858000	-2.955257000	-0.524609000
6	2.921455000	-2.033213000	1.413791000
1	3.556688000	-2.832418000	1.827551000
1	3.598806000	-1.184742000	1.236746000
6	1.887753000	-1.706955000	2.484338000
1	2.403369000	-1.532440000	3.446797000
1	1.231883000	-2.579055000	2.618619000
6	1.810737000	0.717855000	2.179719000
1	2.364097000	0.841015000	3.128189000
1	2.527208000	0.714495000	1.354106000
1	1.133509000	1.566811000	2.045543000
6	-0.003343000	-0.402035000	3.284235000
1	0.457395000	-0.577043000	4.271836000
1	-0.365342000	0.633467000	3.277690000
6	-1.148734000	-1.363506000	3.066429000
1	-1.894054000	-1.253097000	3.874273000
1	-0.788614000	-2.400317000	3.097868000
7	-1.783738000	-1.142538000	1.744286000
7	-1.280109000	-1.285754000	-1.355329000
7	1.538730000	-1.620616000	-0.687749000
7	1.020623000	-0.529635000	2.212381000
8	-0.029129000	1.202367000	-0.032181000
1	-3.779623000	-3.282868000	-0.072858000
1	-3.849163000	-1.537474000	-0.201275000
1	-3.426732000	-2.413347000	2.128585000
1	0.099883000	-0.611156000	-2.782074000
1	-0.700244000	-2.127114000	-3.230528000
1	1.650337000	-2.570422000	-2.595540000
6	-2.591490000	0.092854000	1.767673000
1	-3.104410000	0.234501000	0.812803000

1	-3.349071000	0.046512000	2.570550000
1	-1.945562000	0.964853000	1.921425000
8	0.024651000	4.702865000	-2.314944000
15	0.178831000	3.300349000	-1.747941000
8	-1.094041000	2.956870000	-0.826268000
8	1.435919000	3.367103000	-0.656528000
8	0.465279000	2.158717000	-2.732881000
1	1.357244000	2.521711000	-0.167487000
8	-0.575891000	3.362004000	1.812553000
1	-0.834597000	3.207502000	0.869557000
1	0.360242000	3.593085000	1.714014000

^{4}TS2

E(UB3LYP) = -1761.57955235 hartree

29	53.761889000	2.645886000	4.412766000
6	51.322983000	1.119082000	5.310006000
1	51.991790000	0.246805000	5.363710000
6	50.727395000	1.193240000	3.911681000
6	51.746339000	1.174211000	2.779152000
1	51.218386000	1.030060000	1.818649000
1	52.413358000	0.311415000	2.918290000
6	51.806554000	3.580382000	2.312313000
1	51.259873000	3.419772000	1.365645000
1	51.082109000	3.809046000	3.098299000
1	52.467602000	4.446274000	2.196837000
6	53.609088000	2.170827000	1.570179000
6	54.783892000	1.363946000	2.075071000
1	54.454829000	0.368925000	2.403006000
6	56.219265000	3.192221000	2.774374000
1	55.600956000	3.880454000	2.189838000
1	56.619914000	3.750871000	3.623532000
1	57.061433000	2.845515000	2.148821000
6	56.250858000	1.055775000	3.974687000
1	55.606436000	0.189505000	4.188848000

1	57.056909000	0.709229000	3.301422000
6	56.856313000	1.555203000	5.279486000
1	57.531319000	0.760433000	5.634659000
1	57.498168000	2.433790000	5.117066000
6	55.854526000	1.795215000	6.402183000
1	56.400353000	1.955503000	7.350192000
1	55.244231000	0.889385000	6.526996000
6	55.650642000	4.224835000	6.250025000
1	56.162420000	4.347329000	7.221535000
1	56.396936000	4.276719000	5.452789000
1	54.942400000	5.053178000	6.118150000
6	53.912724000	2.942730000	7.307164000
1	54.394247000	2.696781000	8.269107000
1	53.516889000	3.961578000	7.398955000
6	52.795592000	1.967646000	7.019004000
1	52.065433000	1.971226000	7.847678000
1	53.192076000	0.946865000	6.936736000
7	52.123242000	2.295333000	5.737216000
7	52.603522000	2.382823000	2.647657000
7	55.420794000	2.038076000	3.234247000
7	54.921925000	2.940253000	6.215079000
8	53.768450000	4.857859000	4.231993000
1	50.110722000	0.288764000	3.788592000
1	50.028886000	2.037103000	3.813761000
1	50.515937000	0.959714000	6.049383000
1	53.937466000	3.158780000	1.226135000
1	53.140455000	1.663845000	0.709523000
1	55.520851000	1.217203000	1.266024000
6	51.287063000	3.501141000	5.892918000
1	50.722895000	3.698852000	4.977659000
1	50.570984000	3.369833000	6.723428000
1	51.915277000	4.377858000	6.082632000
8	55.023856000	8.136156000	1.079589000
15	54.508609000	6.833707000	1.683440000
8	53.079908000	6.987428000	2.361639000

8	55.524323000	6.522276000	2.979000000
8	54.533116000	5.602808000	0.758546000
1	55.089025000	5.824233000	3.518763000
8	52.965764000	6.784286000	4.778979000
1	53.014105000	6.911121000	3.644063000
1	53.861603000	7.047220000	5.050572000

^{4}INT2

E(UB3LYP) = -1761.58641376 hartree

29	-0.118667000	-0.870185000	0.608985000
6	-2.619015000	-2.352638000	1.521702000
1	-1.981931000	-3.246517000	1.602044000
6	-3.194402000	-2.285418000	0.114304000
6	-2.162723000	-2.361416000	-1.004357000
1	-2.683802000	-2.516566000	-1.967158000
1	-1.524014000	-3.240103000	-0.834342000
6	-2.022468000	0.024227000	-1.549883000
1	-2.586287000	-0.163831000	-2.481520000
1	-2.726788000	0.322848000	-0.768982000
1	-1.328994000	0.853998000	-1.721527000
6	-0.251747000	-1.467438000	-2.210992000
6	0.885637000	-2.298195000	-1.659778000
1	0.514947000	-3.266254000	-1.297465000
6	2.392018000	-0.509294000	-1.020216000
1	1.810751000	0.181647000	-1.638109000
1	2.812213000	0.064709000	-0.190765000
1	3.223352000	-0.920469000	-1.621558000
6	2.320194000	-2.591647000	0.262408000
1	1.640044000	-3.423869000	0.500426000
1	3.120867000	-2.995544000	-0.385777000
6	2.929715000	-2.066674000	1.555011000
1	3.576532000	-2.868229000	1.945687000
1	3.599884000	-1.214590000	1.367998000
6	1.925096000	-1.753849000	2.656081000

1	2.466581000	-1.568925000	3.602248000
1	1.291271000	-2.638317000	2.812416000
6	1.775040000	0.674016000	2.413141000
1	2.274801000	0.824548000	3.387053000
1	2.533287000	0.681193000	1.625186000
1	1.082321000	1.507376000	2.237354000
6	0.000081000	-0.538691000	3.492512000
1	0.464803000	-0.760233000	4.468596000
1	-0.377139000	0.490085000	3.545051000
6	-1.133799000	-1.501084000	3.221831000
1	-1.870544000	-1.459176000	4.044362000
1	-0.757401000	-2.531729000	3.172542000
7	-1.785788000	-1.195512000	1.926780000
7	-1.266941000	-1.183772000	-1.159449000
7	1.538071000	-1.605583000	-0.521380000
7	1.022519000	-0.596171000	2.413268000
8	0.063403000	1.232991000	0.219388000
1	-3.842580000	-3.168671000	-0.001131000
1	-3.859807000	-1.418376000	-0.009060000
1	-3.441142000	-2.467798000	2.253412000
1	0.118742000	-0.505086000	-2.582427000
1	-0.723917000	-1.987031000	-3.062603000
1	1.622522000	-2.507562000	-2.455685000
6	-2.584678000	0.040447000	2.035289000
1	-3.140795000	0.217012000	1.110931000
1	-3.307586000	-0.034623000	2.867666000
1	-1.931710000	0.905677000	2.190812000
8	1.416797000	4.245213000	-3.246827000
15	0.794097000	3.087381000	-2.495099000
8	-0.706866000	3.526035000	-2.000786000
8	1.610635000	2.912420000	-1.077792000
8	0.706187000	1.732408000	-3.199490000
1	1.141000000	2.226124000	-0.519804000
8	-1.183218000	2.916556000	0.519603000
1	-0.922005000	3.237446000	-1.057133000

1	-0.357511000	3.358523000	0.785649000

^{2}ISC

E(UB3LYP) = -1761.58524597 hartree

29	-0.058404000	-0.901513000	0.565937000
6	-2.550295000	-2.378497000	1.526014000
1	-1.901870000	-3.262502000	1.624786000
6	-3.131468000	-2.350459000	0.119199000
6	-2.104833000	-2.456468000	-1.001790000
1	-2.630108000	-2.636708000	-1.958010000
1	-1.468690000	-3.332280000	-0.810743000
6	-1.966268000	-0.092874000	-1.625605000
1	-2.516708000	-0.310276000	-2.558920000
1	-2.685012000	0.215595000	-0.861730000
1	-1.273650000	0.735338000	-1.805654000
6	-0.189559000	-1.600843000	-2.231090000
6	0.925922000	-2.448860000	-1.660243000
1	0.535375000	-3.406804000	-1.292331000
6	2.478637000	-0.699188000	-1.035034000
1	1.926128000	-0.004967000	-1.675305000
1	2.902208000	-0.118549000	-0.212070000
1	3.307007000	-1.148322000	-1.613242000
6	2.321343000	-2.746507000	0.292725000
1	1.610268000	-3.545901000	0.552156000
1	3.110929000	-3.198965000	-0.337192000
6	2.943394000	-2.201501000	1.571034000
1	3.553054000	-3.014945000	1.995601000
1	3.649891000	-1.385504000	1.359580000
6	1.944573000	-1.809246000	2.651688000
1	2.486854000	-1.616405000	3.595912000
1	1.274778000	-2.661743000	2.831132000
6	1.893519000	0.616291000	2.346942000
1	2.399534000	0.761351000	3.318477000
1	2.651500000	0.583336000	1.558567000

1	1.233635000	1.470157000	2.153042000
6	0.062937000	-0.496943000	3.436154000
1	0.519770000	-0.676019000	4.424899000
1	-0.298270000	0.539045000	3.432405000
6	-1.085827000	-1.454216000	3.210058000
1	-1.822241000	-1.360293000	4.028575000
1	-0.727641000	-2.492284000	3.216144000
7	-1.731656000	-1.201978000	1.901077000
7	-1.208433000	-1.284460000	-1.193284000
7	1.582313000	-1.756458000	-0.525529000
7	1.091678000	-0.622937000	2.368272000
8	0.156770000	1.164748000	0.142571000
1	-3.778519000	-3.237727000	0.030661000
1	-3.798455000	-1.487780000	-0.024544000
1	-3.369416000	-2.487105000	2.262413000
1	0.204299000	-0.649612000	-2.608245000
1	-0.665302000	-2.121091000	-3.080757000
1	1.664926000	-2.680572000	-2.448072000
6	-2.541650000	0.028601000	1.967381000
1	-3.051624000	0.204999000	1.016884000
1	-3.302360000	-0.047420000	2.765727000
1	-1.899518000	0.895022000	2.154852000
8	1.791681000	3.987372000	-3.297637000
15	1.049899000	2.953430000	-2.479050000
8	-0.384362000	3.603340000	-2.017660000
8	1.843634000	2.765988000	-1.051048000
8	0.798968000	1.575004000	-3.091449000
1	1.317623000	2.117809000	-0.494413000
8	-1.095756000	2.703049000	0.385526000
1	-0.716239000	3.208444000	-1.156226000
1	-0.339482000	3.205080000	0.737992000

^{2}PC2

E(UB3LYP) = -1761.63934174 hartree

29	53.917155000	2.747564000	4.387501000
6	51.599482000	0.968892000	5.290452000
1	52.337477000	0.152088000	5.277943000
6	50.957722000	1.071843000	3.913533000
6	51.944747000	1.143182000	2.755446000
1	51.401358000	0.991376000	1.804273000
1	52.656133000	0.312008000	2.857156000
6	51.863279000	3.540937000	2.291088000
1	51.312383000	3.335499000	1.355101000
1	51.140332000	3.737376000	3.087123000
1	52.476683000	4.436085000	2.155802000
6	53.735896000	2.247533000	1.541669000
6	54.917276000	1.421253000	1.994063000
1	54.604204000	0.399986000	2.247031000
6	56.384630000	3.167534000	2.802772000
1	55.800637000	3.862356000	2.191565000
1	56.730680000	3.719686000	3.679855000
1	57.263301000	2.826602000	2.224667000
6	56.339482000	0.964473000	3.878946000
1	55.657482000	0.119296000	4.059450000
1	57.123112000	0.611609000	3.181600000
6	56.979378000	1.368324000	5.197551000
1	57.601299000	0.517626000	5.518989000
1	57.676876000	2.208426000	5.064150000
6	55.988587000	1.628277000	6.324643000
1	56.533521000	1.705190000	7.283977000
1	55.319906000	0.759887000	6.400769000
6	55.973595000	4.060876000	6.294488000
1	56.418011000	4.123979000	7.304217000
1	56.787806000	4.049946000	5.563493000
1	55.349707000	4.939086000	6.103759000
6	54.129634000	2.880820000	7.263682000
1	54.610257000	2.676544000	8.237195000
1	53.732731000	3.903345000	7.307856000
6	53.006055000	1.895814000	7.024878000

1	52.295834000	1.928213000	7.870575000
1	53.394864000	0.870655000	6.975564000
7	52.309008000	2.184734000	5.749842000
7	52.732734000	2.398421000	2.629401000
7	55.548854000	2.016438000	3.196786000
7	55.143627000	2.843910000	6.178863000
8	53.881630000	4.939848000	4.386898000
1	50.376247000	0.147567000	3.767646000
1	50.223467000	1.889991000	3.870521000
1	50.828311000	0.705165000	6.039751000
1	54.059767000	3.254274000	1.251952000
1	53.265867000	1.783158000	0.656476000
1	55.653317000	1.338168000	1.174484000
6	51.376170000	3.310156000	5.946122000
1	50.893557000	3.586337000	5.005586000
1	50.595002000	3.041701000	6.681218000
1	51.920867000	4.191224000	6.301814000
8	55.481573000	7.888103000	0.999306000
15	54.770401000	6.718104000	1.647987000
8	53.276398000	7.241432000	2.119353000
8	55.487985000	6.381049000	3.070862000
8	54.599939000	5.436613000	0.829645000
1	54.881252000	5.745531000	3.626716000
8	52.645701000	5.710560000	4.263605000
1	52.951731000	6.706596000	2.888049000
1	52.654260000	6.237493000	5.080131000

^{2}5a

E(UB3LYP) = -1118.21007329 hartree

29	5.868397000	2.004141000	0.642256000
6	3.145816000	0.423687000	1.418974000
1	3.724542000	-0.510173000	1.339730000
6	2.622586000	0.787057000	0.033800000
6	3.679690000	0.832672000	-1.065430000

1	3.172869000	0.799862000	-2.048200000
1	4.287857000	-0.078356000	-0.987412000
6	3.872246000	3.253875000	-1.293654000
1	3.276459000	3.203864000	-2.223416000
1	3.201775000	3.456296000	-0.453925000
1	4.574086000	4.091909000	-1.372621000
6	5.573050000	1.822354000	-2.208283000
6	6.574149000	0.720683000	-1.918319000
1	6.067460000	-0.247041000	-1.815918000
6	8.372154000	1.985911000	-0.937814000
1	7.947209000	2.877558000	-1.411659000
1	8.855411000	2.311458000	-0.013136000
1	9.138799000	1.553281000	-1.607342000
6	7.864995000	-0.248637000	-0.103801000
1	7.034238000	-0.966054000	-0.018110000
1	8.591898000	-0.671716000	-0.824300000
6	8.531463000	-0.096442000	1.257710000
1	8.978117000	-1.074087000	1.499762000
1	9.375880000	0.607107000	1.214921000
6	7.585032000	0.233930000	2.405945000
1	8.117071000	0.093310000	3.365411000
1	6.760253000	-0.491038000	2.386176000
6	8.010526000	2.629050000	2.652203000
1	8.486591000	2.486109000	3.639147000
1	8.789992000	2.589322000	1.884929000
1	7.532743000	3.613819000	2.611080000
6	5.982112000	1.682343000	3.512366000
1	6.428789000	1.309359000	4.451958000
1	5.755296000	2.746040000	3.660634000
6	4.704480000	0.916030000	3.214961000
1	4.045367000	0.963002000	4.103896000
1	4.925957000	-0.147013000	3.051057000
7	4.026082000	1.438028000	2.018529000
7	4.612804000	1.997973000	-1.088731000
7	7.306410000	1.000748000	-0.663855000

7	6.984817000	1.596180000	2.416021000
8	5.658157000	3.959149000	1.047871000
1	1.918536000	-0.008990000	-0.257947000
1	2.023402000	1.709198000	0.058171000
1	2.289581000	0.217847000	2.094645000
1	6.092466000	2.780144000	-2.348264000
1	5.032151000	1.602751000	-3.146601000
1	7.274645000	0.623173000	-2.767031000
6	3.280622000	2.656879000	2.356892000
1	2.836136000	3.107806000	1.463550000
1	2.466274000	2.438802000	3.076849000
1	3.952416000	3.402492000	2.797479000
8	6.709095000	4.611052000	0.266239000
1	6.424238000	4.453745000	-0.649552000

^{2}**5b**

E(UB3LYP) = -1118.19971484 hartree

29	5.772908000	1.841298000	0.692198000
6	3.159808000	0.438300000	1.395944000
1	3.733324000	-0.496497000	1.302140000
6	2.575627000	0.791519000	0.035688000
6	3.588001000	0.831191000	-1.103401000
1	3.050369000	0.875308000	-2.068639000
1	4.159985000	-0.107119000	-1.091408000
6	3.873051000	3.253342000	-1.253692000
1	3.355422000	3.271568000	-2.230051000
1	3.126494000	3.419870000	-0.471604000
1	4.621286000	4.055994000	-1.211837000
6	5.548171000	1.809769000	-2.172948000
6	6.575431000	0.744610000	-1.861201000
1	6.090497000	-0.230577000	-1.724554000
6	8.322337000	2.112944000	-0.887907000
1	7.826581000	3.067673000	-1.100317000
1	8.957910000	2.269703000	-0.011719000

1	8.969442000	1.819121000	-1.734409000
6	7.932925000	-0.170043000	-0.084105000
1	7.137558000	-0.929701000	-0.040049000
1	8.689513000	-0.527297000	-0.808503000
6	8.566870000	-0.049878000	1.295243000
1	9.034150000	-1.024513000	1.508856000
1	9.393502000	0.675586000	1.301529000
6	7.589690000	0.212470000	2.434498000
1	8.112912000	0.076053000	3.398901000
1	6.790011000	-0.539896000	2.389297000
6	7.939486000	2.622466000	2.665127000
1	8.470599000	2.469148000	3.621597000
1	8.675251000	2.639948000	1.856799000
1	7.426282000	3.589173000	2.677662000
6	5.958350000	1.614673000	3.566413000
1	6.398564000	1.194952000	4.487898000
1	5.746202000	2.673416000	3.763746000
6	4.683301000	0.882286000	3.215467000
1	3.970856000	0.939546000	4.057716000
1	4.886572000	-0.181998000	3.040525000
7	4.077048000	1.443743000	1.984337000
7	4.559574000	1.957360000	-1.071379000
7	7.314792000	1.067636000	-0.617309000
7	6.943274000	1.554012000	2.454666000
8	5.976232000	4.090358000	0.835080000
1	1.859727000	-0.008426000	-0.211946000
1	1.978125000	1.714056000	0.080363000
1	2.338643000	0.249896000	2.113635000
1	6.038998000	2.782785000	-2.301423000
1	5.029046000	1.571138000	-3.118150000
1	7.275563000	0.638861000	-2.708880000
6	3.379019000	2.702099000	2.307718000
1	2.989070000	3.178950000	1.404789000
1	2.537199000	2.515260000	2.999030000
1	4.076478000	3.405169000	2.779499000

8	6.426022000	4.856419000	-0.349708000
1	5.067219000	4.402339000	0.965416000

^{3}**6a**

E(UB3LYP) = -1118.04421493 hartree

29	5.762683000	1.741581000	0.688516000
6	3.152859000	0.443758000	1.382410000
1	3.708445000	-0.500980000	1.283021000
6	2.564739000	0.809686000	0.027629000
6	3.570242000	0.827590000	-1.117470000
1	3.030215000	0.880376000	-2.080424000
1	4.128466000	-0.118864000	-1.106143000
6	3.884400000	3.236712000	-1.274958000
1	3.323535000	3.237746000	-2.226767000
1	3.183457000	3.442839000	-0.462105000
1	4.620337000	4.046483000	-1.308440000
6	5.538620000	1.769763000	-2.200929000
6	6.566199000	0.709498000	-1.879836000
1	6.082464000	-0.264659000	-1.733584000
6	8.351611000	2.041843000	-0.933941000
1	7.942268000	2.866576000	-1.529649000
1	8.771096000	2.464799000	-0.017872000
1	9.164189000	1.572597000	-1.516209000
6	7.881809000	-0.195335000	-0.067462000
1	7.054820000	-0.914326000	0.034664000
1	8.590910000	-0.610158000	-0.807529000
6	8.577495000	-0.048315000	1.277658000
1	9.069893000	-1.012119000	1.482779000
1	9.388897000	0.693019000	1.234202000
6	7.643926000	0.211427000	2.451903000
1	8.211328000	0.129135000	3.396796000
1	6.870854000	-0.569554000	2.465451000
6	7.913211000	2.632491000	2.641443000
1	8.439781000	2.515520000	3.605244000

1	8.658680000	2.649080000	1.841595000
1	7.376912000	3.587535000	2.639727000
6	5.966077000	1.572279000	3.566689000
1	6.406185000	1.134834000	4.479047000
1	5.754826000	2.626685000	3.784855000
6	4.694215000	0.846198000	3.196483000
1	3.973439000	0.887293000	4.031745000
1	4.901212000	-0.213544000	2.999065000
7	4.094778000	1.431197000	1.970001000
7	4.563435000	1.938428000	-1.090382000
7	7.299539000	1.051066000	-0.633389000
7	6.950037000	1.528074000	2.451144000
8	5.815318000	4.264252000	1.030791000
1	1.832523000	0.023153000	-0.214397000
1	1.985308000	1.743377000	0.075907000
1	2.336132000	0.271542000	2.107766000
1	6.027752000	2.739665000	-2.362014000
1	5.009439000	1.515157000	-3.135836000
1	7.271451000	0.596498000	-2.721011000
6	3.422566000	2.703327000	2.300131000
1	3.043475000	3.191382000	1.399415000
1	2.577832000	2.524155000	2.988772000
1	4.131896000	3.390845000	2.773659000
8	6.628756000	4.804975000	0.154795000
1	6.632657000	4.203870000	-0.625116000

^{3}**6b**

E(UB3LYP) = -1118.04298523 hartree

29	5.748976000	1.685480000	0.699614000
6	3.117980000	0.467404000	1.370848000
1	3.647809000	-0.489420000	1.249086000
6	2.524539000	0.873855000	0.029845000
6	3.510793000	0.862000000	-1.130356000
1	2.957703000	0.954345000	-2.082862000

1	4.023383000	-0.110412000	-1.142965000
6	3.957980000	3.253738000	-1.301321000
1	3.385607000	3.281873000	-2.245960000
1	3.277980000	3.505985000	-0.481508000
1	4.748364000	4.011480000	-1.354660000
6	5.522890000	1.677465000	-2.216823000
6	6.548520000	0.639203000	-1.826636000
1	6.056309000	-0.317569000	-1.607443000
6	8.228872000	2.146742000	-0.926487000
1	7.680432000	3.023139000	-1.287644000
1	8.786254000	2.448390000	-0.035789000
1	8.947479000	1.821462000	-1.699740000
6	7.993326000	-0.137595000	-0.063275000
1	7.245408000	-0.941834000	0.001341000
1	8.754106000	-0.453989000	-0.800510000
6	8.642716000	0.034738000	1.301586000
1	9.187493000	-0.900530000	1.506594000
1	9.408440000	0.824122000	1.291213000
6	7.664935000	0.223533000	2.453756000
1	8.206496000	0.124068000	3.412049000
1	6.913157000	-0.577929000	2.417855000
6	7.872706000	2.645815000	2.682636000
1	8.458193000	2.488577000	3.605922000
1	8.565011000	2.742078000	1.841861000
1	7.307725000	3.578434000	2.771708000
6	5.947260000	1.518350000	3.588277000
1	6.381475000	1.044989000	4.485253000
1	5.731769000	2.561641000	3.849609000
6	4.679194000	0.803016000	3.182320000
1	3.949035000	0.818137000	4.010214000
1	4.891430000	-0.249710000	2.953286000
7	4.089943000	1.422068000	1.966104000
7	4.559325000	1.920270000	-1.108299000
7	7.289338000	1.053569000	-0.607691000
7	6.936383000	1.521863000	2.476925000

8	5.930426000	4.247432000	0.960400000
1	1.755341000	0.122460000	-0.208987000
1	1.987266000	1.831431000	0.096172000
1	2.305880000	0.303898000	2.103048000
1	6.012058000	2.630179000	-2.454229000
1	4.986046000	1.357317000	-3.126066000
1	7.251893000	0.464599000	-2.659531000
6	3.445935000	2.705523000	2.308799000
1	3.079114000	3.207275000	1.408819000
1	2.596339000	2.540506000	2.994537000
1	4.169035000	3.372780000	2.791908000
8	6.698081000	4.895854000	0.114632000
1	5.011117000	4.577975000	0.839202000

^{3}**7**

E(UB3LYP) = -1117.58440642 hartree

29	6.629293000	4.112723000	3.221787000
6	4.190907000	2.580680000	4.090440000
1	4.846429000	1.696908000	4.095891000
6	3.562974000	2.715659000	2.710299000
6	4.555053000	2.740652000	1.553729000
1	4.006255000	2.627130000	0.600524000
1	5.223782000	1.873938000	1.649261000
6	4.585756000	5.148365000	1.146495000
1	4.050702000	5.003735000	0.190538000
1	3.848541000	5.338933000	1.931414000
1	5.235350000	6.026191000	1.079383000
6	6.392515000	3.791791000	0.354252000
6	7.545189000	2.910584000	0.775590000
1	7.186997000	1.903768000	1.025870000
6	9.096965000	4.587877000	1.583157000
1	8.507417000	5.351604000	1.063639000
1	9.543412000	5.061581000	2.460973000
1	9.904076000	4.236377000	0.915612000

6	8.987050000	2.366082000	2.625166000
1	8.275672000	1.540699000	2.780433000
1	9.754644000	2.008883000	1.913310000
6	9.644813000	2.697892000	3.956380000
1	10.234631000	1.811244000	4.239061000
1	10.374331000	3.514461000	3.853458000
6	8.673700000	2.952823000	5.102692000
1	9.231955000	2.986706000	6.056669000
1	7.975907000	2.105755000	5.163471000
6	8.734139000	5.391001000	5.106194000
1	9.228501000	5.406129000	6.094474000
1	9.512280000	5.382993000	4.337570000
1	8.126233000	6.294075000	4.983528000
6	6.869043000	4.255914000	6.094362000
1	7.340686000	3.977553000	7.053296000
1	6.538441000	5.298254000	6.186860000
6	5.688674000	3.353662000	5.816532000
1	4.970860000	3.399821000	6.654273000
1	6.016059000	2.309760000	5.728813000
7	5.018801000	3.728144000	4.545330000
7	5.404127000	3.958962000	1.452311000
7	8.225668000	3.463379000	1.974334000
7	7.868920000	4.198431000	4.996294000
8	6.691642000	6.389659000	2.951655000
1	2.939027000	1.819360000	2.565627000
1	2.866170000	3.565441000	2.664648000
1	3.398266000	2.406137000	4.841901000
1	6.758120000	4.790160000	0.082286000
1	5.901528000	3.370796000	-0.540761000
1	8.262687000	2.801064000	-0.056379000
6	4.206086000	4.944345000	4.741992000
1	3.670388000	5.202338000	3.824931000
1	3.467104000	4.789678000	5.548209000
1	4.850977000	5.794655000	4.988871000
8	6.450563000	7.248025000	3.936757000

^{4}8

E(UB3LYP) = -1117.44875888 hartree

29	6.648199000	3.800462000	3.253694000
6	3.966516000	2.728622000	4.074005000
1	4.414538000	1.723966000	4.069205000
6	3.398559000	3.017755000	2.691917000
6	4.382680000	2.842884000	1.543323000
1	3.830809000	2.877617000	0.586260000
1	4.850796000	1.849355000	1.617389000
6	4.980976000	5.205327000	1.222634000
1	4.378429000	5.237680000	0.297098000
1	4.363339000	5.554209000	2.055088000
1	5.830279000	5.888803000	1.117360000
6	6.415435000	3.449064000	0.365235000
6	7.555498000	2.618952000	0.909798000
1	7.171059000	1.677219000	1.326677000
6	8.898302000	4.572438000	1.510889000
1	8.143670000	5.315111000	1.229061000
1	9.523898000	5.012858000	2.292786000
1	9.535841000	4.347959000	0.637530000
6	9.226284000	2.416864000	2.640611000
1	8.697420000	1.467429000	2.811645000
1	10.034380000	2.217564000	1.913229000
6	9.822414000	2.889139000	3.958896000
1	10.576131000	2.139271000	4.246729000
1	10.378715000	3.830645000	3.838864000
6	8.834303000	2.973981000	5.115132000
1	9.392093000	3.121092000	6.058001000
1	8.291861000	2.020022000	5.198534000
6	8.416581000	5.394420000	5.036123000
1	8.998936000	5.546030000	5.962580000
1	9.081968000	5.536044000	4.180079000
1	7.621704000	6.146832000	4.984340000

6	6.858527000	3.919103000	6.163415000
1	7.358402000	3.458072000	7.029827000
1	6.547009000	4.922724000	6.476530000
6	5.659068000	3.095016000	5.757581000
1	4.930603000	3.041649000	6.586161000
1	5.971167000	2.069101000	5.514144000
7	5.018495000	3.666255000	4.545482000
7	5.486041000	3.840232000	1.466158000
7	8.249387000	3.340140000	2.007539000
7	7.809093000	4.049374000	5.020653000
8	7.257220000	7.335947000	2.663641000
1	2.592788000	2.285138000	2.525825000
1	2.911097000	4.003231000	2.650598000
1	3.151291000	2.722696000	4.820587000
1	6.795681000	4.359551000	-0.113193000
1	5.875431000	2.886255000	-0.412300000
1	8.269084000	2.368208000	0.105011000
6	4.467508000	5.009959000	4.821357000
1	3.853934000	5.347474000	3.981171000
1	3.837025000	4.991325000	5.727663000
1	5.276238000	5.737226000	4.957271000
8	6.223505000	7.550889000	3.232082000
